# Branched-chain amino acids in muscle growth: mechanisms, physiological functions, and applications

**DOI:** 10.1186/s40104-025-01300-y

**Published:** 2025-12-03

**Authors:** Shuyong Xu, Guangyong Zhao, Mark D. Hanigan, Gonzalo Cantalapiedra-Hijar, Mengmeng Li

**Affiliations:** 1 State Key Laboratory of Animal Nutrition and Feeding, College of Animal Science and Technology, China Agricultural University, Beijing, 100193 P. R. China; 2 School of Animal Sciences, Virginia Tech, Blacksburg, VA 24060 USA; 3 Université Clermont Auvergne, INRAE, VetAgro Sup, UMR Herbivores, Saint-Genès-Champanelle, 63122 France

**Keywords:** Animal nutrition, Branched-chain amino acid, Metabolic regulation, Signaling mechanism, Skeletal muscle

## Abstract

Skeletal muscle accounts for approximately 40% of body mass and 50%–75% of whole-body protein, playing a central role in meat production and quality. Efficient protein synthesis in skeletal muscle relies on an adequate supply of nutrient substrates and a balanced amino acid profile. Branched-chain amino acids (BCAA), including leucine (Leu), isoleucine (Ile), and valine (Val), are the most abundant essential amino acids in skeletal muscle and contribute to both protein synthesis and oxidative energy production. Additionally, BCAA function as signaling molecules that regulate gene expression and protein phosphorylation cascades, which significantly influence physiological processes, such as protein synthesis and degradation, glucose and lipid metabolism, and cell apoptosis and autophagy. These processes are primarily mediated through the PI3K/AKT/AMPK/mTOR signaling pathways. This review summarizes BCAA transporters and catabolic metabolism, their role as signaling molecules in regulating protein metabolism and glucose and lipid equilibrium, and applications in animal production. These findings offer both theoretical insights and practical guidelines for the precise regulation of feed efficiency and production performance through tailored dietary BCAA supplementations.

**Graphical Abstract:**

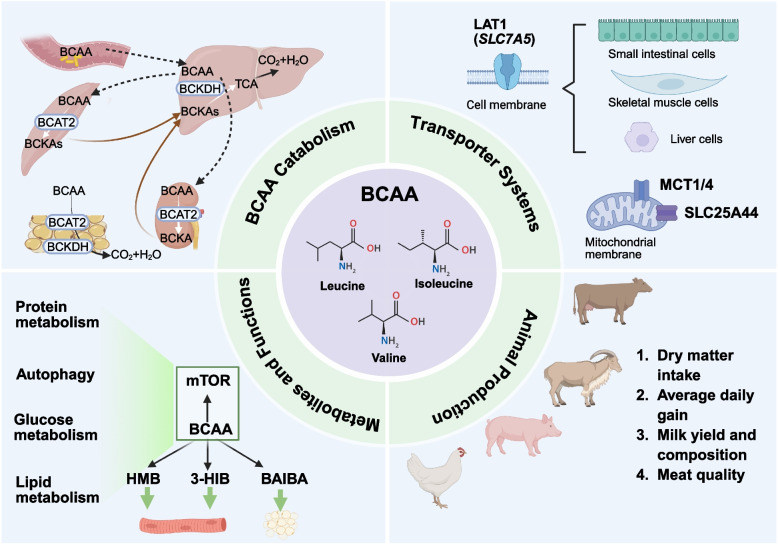

## Introduction

Meat yield is a determinant of production efficiency in animal husbandry and together with meat quality governs the economic benefits of farming. Skeletal muscle contributes approximately 40% of total body weight and 50%–75% of whole-body protein in livestock, serving not only as a major structural component but also as an essential metabolic tissue involved in various biochemical processes and physiological functions [1, 2]. Understanding the metabolic mechanisms of skeletal muscle and its regulatory factors is essential for optimizing production efficiency in livestock systems. Recent studies have increasingly examined the influence of nutrients on skeletal muscle metabolic pathways, growth, and meat quality, with particular emphasis on the roles of essential amino acids (EAA) and their metabolites [3, 4]. Among these, branched-chain amino acids (BCAA) constitute a distinct family of EAA that play critical roles on animal growth, metabolism, and overall health.

Branched-chain amino acids, which include leucine (Leu), isoleucine (Ile), and valine (Val), are essential for protein synthesis, accounting for approximately 20% of the total amino acids (AA) in muscle tissue, 35% of EAA, and 50% of the EAA in milk proteins [5–7]. The side chains of BCAA are nonpolar, branched hydrocarbon chains that are small and hydrophobic, preventing them from forming hydrogen bonds with water molecules. These unique properties enable them to stabilize hydrophobic core regions, promote proper folding, and enhance structural rigidity [8]. Beyond serving as substrates for protein synthesis, BCAA function as signaling molecules that regulate gene expression and protein phosphorylation cascades in livestock [9]. Leucine, in particular, acts as a key activator of the mammalian target of rapamycin (mTOR), stimulating muscle protein synthesis, inhibiting protein degradation, and promoting muscle growth through signaling pathways such as PI3K/AKT/mTOR [10].

Besides BCAA, BCAA metabolites (such as β-hydroxy-β-methylbutyrate and 3-hydroxyisobutyrate) also play vital roles in regulating muscle function, including reducing protein degradation, enhancing muscle protein synthesis efficiency, and modulating energy metabolism [10]. Understanding how BCAA and their metabolites influence muscle growth and metabolism is essential for developing more effective nutritional strategies. This review examines the biochemical pathways and signaling networks through which BCAA and their metabolites regulate protein metabolism, as well as glucose and lipid metabolism. Furthermore, it discusses the practical applications of these findings in animal production, offering a theoretical foundation and technical support for the sustainable development of animal husbandry and the improvement of meat yield and quality.

## Transporters involved in BCAA metabolism

**Fig. 1:**
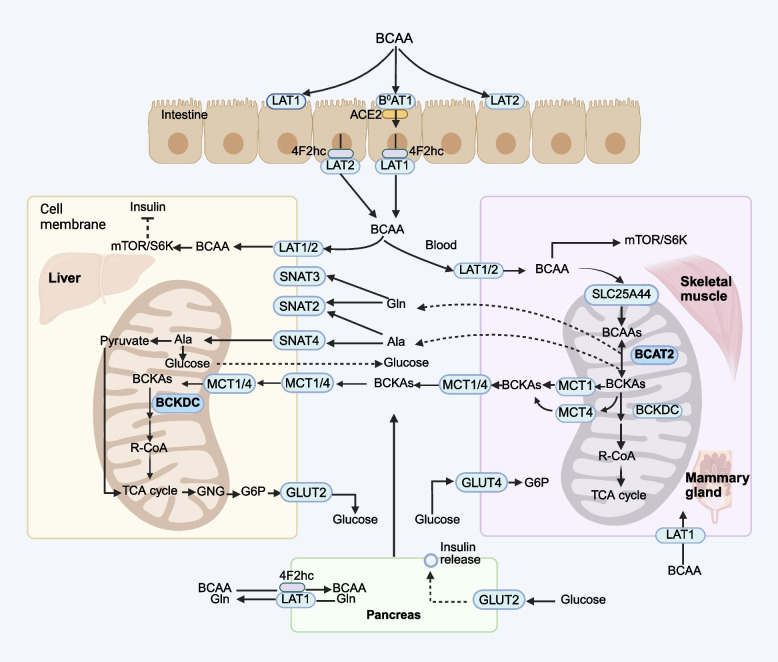
The BCAA transporter systems across cellular and organelle membranes. MCT1, monocarboxylate transporter 1; MCT4, monocarboxylate transporter 4 (SLC16A4); LAT1, L-Type amino acid transporter 1; LAT2, L-Type amino acid transporter 2; GNG, Gluconeogenesis; G6P, Glucose 6-phosphate; GLUT4, glucose transporter 4; GLUT2, glucose transporter 2; SNAT2, sodium-coupled neutral amino acid transporter 2; SNAT3, sodium-coupled neutral amino acid transporter 3; SNAT4, sodium-coupled neutral amino acid transporter 4

AA transporters are membrane-bound proteins that facilitate the movement of AA across cellular and organelle membranes. The expression of transporters varies among different tissues and organelles, where they perform essential functions, such as neurotransmitter transport, acid–base balance, and cellular metabolism. BCAA transport within the body is a complex process regulated by multiple transport proteins (Fig. 1). Although previous work suggested that some AA could be absorbed through the rumen epithelium [11], to the best of our knowledge BCAA transporters have not been reported to be present in the rumen wall. In the small intestine, proteins are digested into peptides and AA, which are subsequently absorbed into the bloodstream through intestinal epithelial cells. The absorption of BCAA in the small intestine primarily relies on system L transporters (LAT1 and LAT2) and system B^0^ transporters [12]. LAT1 and LAT2 are antiporters that mediate the uptake of neutral AA in exchange for another amino acid that was concentrated in the cells in a Na-dependent manner. LAT1 is expressed on both the apical and basolateral membranes of intestinal epithelial cells, facilitating BCAA transport from the intestinal lumen into epithelial cells. From the epithelial cells, BCAA are released into the bloodstream via LAT1-4F2hc complexes located on the basolateral membrane [13]. The LAT1-4F2hc complex, a heterodimer formed by LAT1 and the 4F2hc protein (CD98), promotes cell growth by stimulating mTORC1 signaling and inhibiting integrated stress responses [14] (Fig. 1). LAT2 also contributes to BCAA absorption, particularly in the transport of AA across the intestine wall and into other tissues. Additionally, the B^0^AT1 (SLC6A19) transporter, expressed in the proximal intestine, duodenum, jejunum, and ileum, facilitates BCAA transport from the intestinal lumen into epithelial cells [15]. B^0^AT2 (SLC6A15), a member of the solute carrier 6 (SLC6) gene family of membrane transport proteins, is known as the neurotransmitter transporter. It is expressed in the brain, where it transports BCAA and influences feed intake in animals [15].

After being absorbed into the portal vein, BCAA are primarily catabolized in skeletal muscle, gut, and mammary glands but not the liver. This is because the liver expresses only minimal amounts of BCAA amino transferase (BCAAT), which is responsible for the first step of BCAA catabolism [16]. However, a portion of BCAA are catabolized by the gut tissue [16]. The resulting branched-chain keto acids are released into the portal vein and subsequently cleared by the liver. Any BCAA not metabolized by the gut or liver enter general circulation and are distributed to peripheral tissues. In skeletal muscle, BCAA are mainly transported into cells through LAT1 and LAT2 [17]. In other tissues, such as mammary glands, kidney, fat, and pancreatic cells, BCAA uptake involves multiple transporters from the LAT family (LAT1, LAT2, LAT3, and LAT4). The relative contribution of each transporter depends on the tissue type and regulatory conditions [18, 19]. LAT1 and LAT2 require heterodimerization with the heavy chain 4F2hc (SLC3A2) to be functionally expressed at the plasma membrane, whereas LAT3 and LAT4 operate independently as monomeric transporters. This structural distinction may contribute to differences in their transport efficiency [20, 21]. Within the cell, BCAA are transported into the mitochondria via the SLC25A44 coded transporter for the initial step of metabolism. The resulting branched-chain α-keto acids (BCKA) are then exported from cells by monocarboxylate transporters (MCTs) [22, 23]. The mitochondrial transporter encoded by SLC25A44 has been reported in BCAA catabolism in brown adipocytes [22], suggesting a potential role in other metabolically active tissues such as skeletal muscle and mammary glands. Monocarboxylate transporters, particularly MCT1 (SLC16A1) and MCT4 (SLC16A4), are expressed in both liver and skeletal muscle [23], where they mediate BCKA transport between skeletal muscle and the liver. Notably, MCT1 and MCT4 also facilitate bidirectional BCKA transport across membranes in brain tissue and oocytes [24].

BCAA also serve as essential nitrogen donors for the synthesis of glutamic acid (Glu), alanine (Ala), and glutamine (Gln) in skeletal muscle. Synthesized Gln is transported into hepatocytes via two secondary active transport systems: system A (SLC38A2 or SLC38A4) and system N (SLC38A3 and SLC38A5) [25] (Fig. 1). Glu is transported into mitochondria via SLC25A22 or SLC25A12 encoded transporters [15]. In the liver, SNAT2 (SLC38A2), SNAT3 (SLC38A3), and SNAT4 (SLC38A4) are highly expressed [26]. SNAT2 facilitates the transport of both Ala and Gln, while SNAT3 specifically transports Gln, and SNAT4 primarily mediates Ala transport [27].

## BCAA catabolism in the body

BCAA metabolism in ruminants is initiated by rumen microbial preprocessing, which leads to a net loss of dietary BCAA for the host animal. While dietary proteins are degraded into ammonia, AA, and peptides for microbial protein synthesis, this process is inefficient for BCAA conversion. Consequently, 30%–50% of the absorbed BCAA in ruminants is derived from microbial protein, with the remainder originating from dietary sources that escape ruminal degradation [28, 29]. While microbial protein serves as an important amino acid source, this inherent inefficiency can lead to a BCAA shortfall. This contrasts sharply with monogastric animals (e.g., pigs, poultry), where gastric and pancreatic proteases liberate free BCAA that are absorbed directly into portal circulation without microbial intermediation. This results more efficient absorption but greater dietary AA sensitivity in monogastric species. After being absorbed in the small intestine via sodium-dependent AA transporters, approximately 93% of absorbed Leu bypasses hepatic first-pass metabolism in ruminants and directly enters the peripheral circulation, indicating that only about 7% is utilized by splanchnic tissues [30]. Ruminant liver exhibits limited BCAA clearance (< 5%–10%) due to low activity of key enzymes like BCAT and branched-chain α-keto acid dehydrogenase complex (BCKDC), shifting BCAA catabolism to peripheral tissues (e.g., mammary glands and muscle) [31, 32]. In contrast, monogastric livers exhibit greater BCAA catabolic capacity. Porcine livers clear 9% of portal Leu [33], converting BCAA carbon skeletons into keto acids or TCA cycle intermediates for energy production. Although this represents a measurable contribution, skeletal muscle remains the primary site of BCAA catabolism.

Lobley et al. [31] demonstrated that approximately 25% of whole-body Leu irreversible loss occurs in the portal-drained viscera (PDV), with the mesenteric-drained viscera (MDV) accounting for nearly 40% of this oxidation metabolism. In sheep, net PDV absorption rates of Leu, Ile, and Val (0.56, 0.61, and 0.59 mmol/h, respectively) were consistently lower than those of other EAA such as lysine, histidine, methionine, and phenylalanine, indicating a small proportion of local BCAA utilization by forestomaches, hindgut, pancreas, and spleen [31]. Approximately 93% of absorbed BCAA enter systemic circulation, with mammary tissues receiving around 25% of blood flow of cardiac output and extracting up to 45% of circulating BCAA. These findings suggest mammary and splanchnic tissues utilize comparable amounts of BCAA, though splanchnic uptake involves post-absorptive recycling rather than first-pass metabolism [30, 34].

The complete catabolism of BCAA occurs primarily in mitochondria through the sequential action of two key enzymes BCAT and BCKDC [35]. BCAT exists in two isoforms. BCAT1 is located in the cytoplasm, and BCAT2 resides in the mitochondria. While both isoforms are highly active and reversible, their tissue distributions are mutually exclusive [36]. BCAT1 is predominately expressed in the brain, ovaries, and placenta [37, 38], whereas BCAT2 is found in tissues such as skeletal muscle, kidneys, pancreas, stomach, and colon [39–41]. Despite its widespread distribution, BCAT shows peak expression in skeletal muscle with minimal activity in liver, establishing muscle as the primary site of BCAA transamination [42]. Conversely, BCKDC exhibits a complementary distribution with highest activity in liver, intermediate levels in heart and kidneys, and minimal activity in skeletal muscle [38]. Consequently, most metabolites generated in skeletal muscle following BCAT catalysis undergo further oxidation in the liver.

**Fig. 2:**
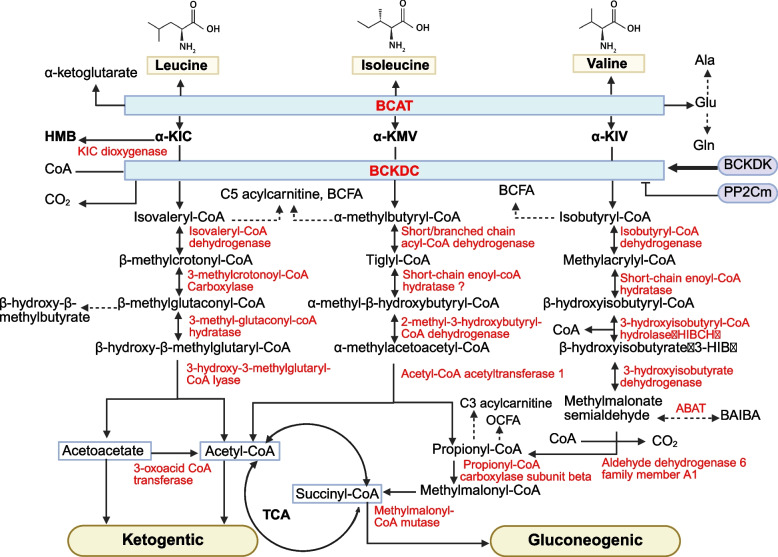
The metabolic pathways of BCAA catabolism modified according to Mann et al. [36]. α-KIC, α-ketoisocaproic acid; α-KMV, α-keto-β-methylvaleric acid; α-KIV, α-ketoisovaleric acid; BAIBA, β-aminoisobutyric acid; OCFA, odd-chain fatty acids; HMB**,** β-hydroxy-β-methylbutyrate; BCAT, branched-chain amino acids transaminase; BCKDC, branched-chain α-keto acid dehydrogenase complex; BCFA, branched-chain fatty acids; BCKDK, BCKDC kinase; PP2Cm, protein phosphatase Mg^2+^/Mn^2+^-dependent 1 K

As illustrated in Fig. 1, BCAA catabolism occurs predominantly in skeletal muscle [43]. Val, Leu, and Ile are converted into α-ketoisovaleric acid (KIV), α-ketoisocaproic acid (KIC), and α-keto-β-methylvaleric acid (KMV), respectively, by BCAT2 in the mitochondria [43, 44] (Fig. 2). Subsequently, BCKA undergo a series of irreversible enzymatic reactions, entering the bloodstream for further catabolism in other tissues. Specifically, α-ketoglutarate (α-KG) acts as the primary acceptor of the amino group to form Glu [42]. Alternatively, the amino group can be transferred to pyruvate to generate Ala or reattached to glutamate to form Gln, which functions as a detoxification pathway for ammonia. These metabolites are released into circulation. Glutamine can activate mTORC1 through a mechanism independent of Rag GTPase, directly stimulating protein synthesis [45]. This underscores the role of elevated glutamine levels in maintaining efficient protein synthesis in skeletal muscle.

The oxidative decarboxylation of BCKA occurs in multiple tissues but primarily in the liver mitochondria [16, 46]. BCKDC is located to the inner mitochondrial membrane and comprises three subunits: a heterotetrameric (α2-β2) branched-chain α-keto acid decarboxylase (E1), a dihydrolipoyl transacylase (E2), and a dihydrolipoamide dehydrogenase (E3). During this process, KMV, KIV, and KIC are converted into α-methylbutyryl-CoA, isobutyryl-CoA, and isovaleryl-CoA, respectively (Fig. 2). The irreversible oxidative decarboxylation catalyzed by BCKDC constitute the rate-limiting step of BCAA degradation. Its activity is regulated by BCKDC kinase (BCKDK) and protein phosphatase Mg^2+^/Mn^2+^-dependent 1 K (PP2Cm) [47, 48]. BCKDK phosphorylates the E1α subunit of BCKDC to inhibit its activity, while PP2Cm dephosphorylates it, activating BCKDC [47, 48]. PP2Cm is a soluble protein localized in the mitochondrial matrix with tissue-specific distribution. It is highly expressed in the brain, liver, kidneys, and heart, but exhibits lower expression in skeletal muscle. Nutritional status, exercise, and hormones modulate BCAA catabolism through BCKDK. Arp et al. [49] demonstrated that reactive nitrogen species inhibit BCKDC activity, thereby suppressing BCAA oxidation in both myotubes and myoblasts.

The next step of BCAA catabolism involves the conversion of α-methylbutyryl-CoA, isobutyryl-CoA, and isopivaloyl-CoA into acetyl-CoA and methylmalonyl-CoA, which enter the tricarboxylic acid (TCA) cycle to generate ATP for cellular energy production (Fig. 2). Following the formation of α-methylbutyryl-CoA, Ile undergoes sequential reactions, including dehydrogenation by branched-chain acyl-CoA dehydrogenase, hydrolysis by short-chain enoyl-CoA hydratase, and cleavage by 2-methyl-3-hydroxybutyryl-CoA dehydratase, generating acetyl-CoA and propionyl-CoA. Propionyl-CoA is subsequently converted to succinyl-CoA via propionyl-CoA β-subunit carboxylase and methylmalonyl-CoA mutase. Similarly, Val metabolism begins with the conversion of isobutyryl-CoA, followed by dehydrogenation by isobutyryl-CoA dehydrogenase, hydration by short-chain enoyl-CoA hydratase, and hydrolysis by 3-hydroxyisobutyryl-CoA hydrolase, leading to the formation of 3-hydroxyisobutyrate (3-HIB), a key regulator of glucose and lipid metabolism [50]. Additionally, 3-HIB is further catalyzed by 3-HIB dehydrogenase to produce methylmalonyl aldehyde, which is then converted into β-aminoisobutyric acid (BAIBA) by 4-aminobutyrate transaminase [51]. BAIBA is then ultimately degraded to succinyl-CoA. Leucine catabolism proceeds through a series of enzymatic reactions catalyzed by isovaleryl-CoA dehydrogenase, 3-methylcrotonyl-CoA carboxylase, 3-methyl-glutaconyl-CoA hydratase, and 3-hydroxy-3-methylglutaryl-CoA lyase, resulting in the production of acetoacetate and acetyl-CoA. Acetoacetate and acetyl-CoA serve as critical intermediates in several metabolic pathways, including the TCA cycle, fatty acid synthesis, and ketogenesis.

Overall, BCAA catabolism provides carbon units to metabolic pathways such as the TCA cycle, ketone body metabolism, and lipid synthesis. Valine is glucogenic (succinyl-CoA is a gluconeogenic precursor), Leu is ketogenic, and Ile is both glucogenic and ketogenic [8]. These AA play a key role in regulating lipid metabolism, glucose metabolism, AA metabolism, and oxidative energy supply in the body.

## Signal pathways of BCAA and their metabolites

### BCAA on the mTOR pathway

BCAA serve dual metabolic roles as both direct substrates for protein synthesis and potent signaling molecules regulating anabolic processes. As structural precursors, they facilitate transcription and translation to support muscle protein accretion. Additionally, BCAA function as key nutrient signals that activate the mechanistic target of rapamycin (mTOR) pathway. The 280 kDa serine/threonine kinase mTOR belongs to the phosphatidylinositol kinase-related kinase family and exists in two functional distinct complexes with unique structures and functions [52]. mTORC1 primarily promotes protein synthesis through S6K1/4E-BP1 activation while suppressing autophagy. mTORC2 serves as downstream effector of insulin/PI3K signaling, primarily regulating glucose metabolism, cytoskeleton organization, cell migration, and adhesion [53].

**Fig. 3:**
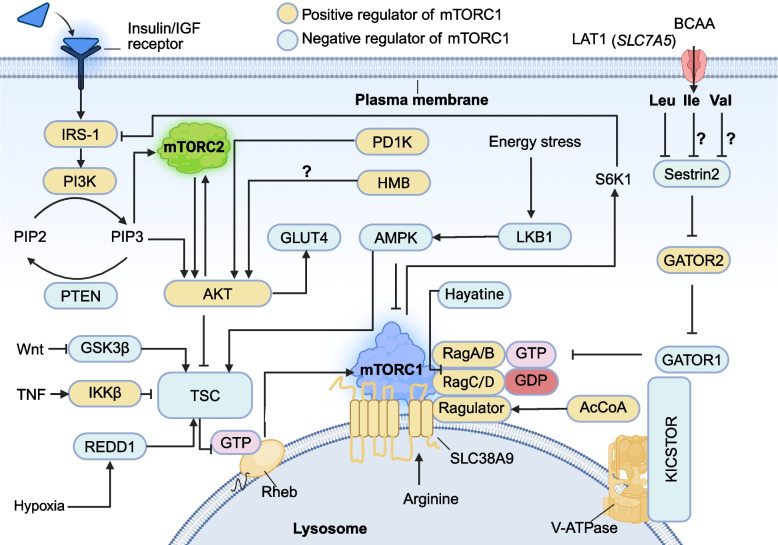
The critical roles of BCAA in modulating the upstream signaling pathways of mTOR. The mTORC1 and mTORC2 complexes are regulated by diverse signaling pathways. Intracellular molecules are activated through various signaling cascades, leading to the formation of either mTORC1 or mTORC2 complexes. IRS-1, insulin receptor substrate 1; LKB1, liver kinase B1; mTORC1/2, mechanistic target of rapamycin complex 1/2; GLUT4, glucose transporter 4; PI3K, phosphoinositide 3-kinase; PIP2, phosphatidylinositol-diphosphate; PIP3, phosphatidylinositol-3,4,5-trisphosphate: PETN, phosphatase and tensin homologue; Rag, ras-related GTP binding; Rheb, ras homolog enriched in brain; TSC, tuberous sclerosis complex; REDD1, transcriptional regulation of DNA damage response 1; AMPK, AMP-activated protein kinase; S6K1, S6 kinase 1; MORG1, WD-domain repeat protein; V-ATPase, vacuolar H-ATPase

As illustrate in Fig. 3, intracellular BCAA activate mTORC1 signaling through the GATOR regulatory network, with Sestrin2 acting as the primary Leu sensor [54]. In the absence of Leu, Sestrin2 binds and inhibits GATOR2. However, upon Leu binding, Sestrin2 dissociates with GATOR2 [55]. Notably, some studies reported that Sestrin1 may play a more prominent role than Sestrin2 in Leu-related mTORC1 activation through similar GATOR2 dissociation mechanisms [56]. The GATOR system operates through coordinated molecular interactions. GATOR2 promotes mTORC1 signaling by inhibiting GATOR1 [57], whereas GATOR1 suppresses mTORC1 by maintaining RagA/B GTPases in their inactive state [58]. The KICSTOR complex spatially organizes this regulatory cascade by tethering GATOR1 to the lysosomal surface, ensuring compartmentalized control of mTORC1 activity [59, 60]. While Leu supplementation stimulates mTORC1 and promotes skeletal muscle regeneration, excessive Leu levels may paradoxically inhibit mTOR in myocytes, reducing cell proliferation and protein synthesis [61].

mTORC1 activation at the lysosomal membrane is regulated by two small GTPases, Rheb and Rag-GTP [62]. Efficient mTORC1 signaling depends on the coordinated input of growth factors and AA, as maximal activation occurs when both Rheb and Rag-GTP are in their active states. At the cellular level, mTORC1 activity exhibits a graded response. Increasing concentrations of growth factors or AA elevate the proportion of active Rheb and Rag-GTP, respectively, leading to a higher probability of mTORC1 activation. Additionally, BCAA promote β-cell proliferation upon their transport into pancreatic cells by the SNAT2 and LAT1 transporters [63]. For this reason, BCAA and insulin/insulin-like growth factor 1 (IGF-1) signaling synergistically enhance mTOC1 activation [53]. Rag GTPases (Rag A/B and Rag C/D) are crucial for mTORC1 signaling by promoting its recruitment to the lysosomal membrane via the Ragulator complex [64, 65]. Under high AA conditions, Rag GTPases facilitate mTORC1 translocation to the lysosomal membrane, where its activation is further potentiated by Rheb-GTP [66] (Fig. 3).

**Fig. 4:**
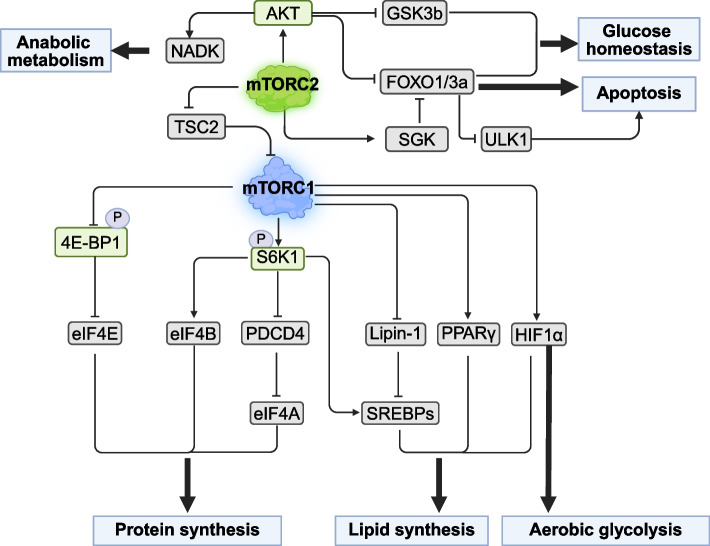
The major downstream pathways of mTORC1 signaling regulate protein synthesis, lipid synthesis, aerobic glycolysis, glucose homeostasis, and apoptosis. AKT, protein kinase B; S6K1, ribosomal protein S6 kinase 1; 4EBP1, eukaryotic translation initiation factor 4E-binding protein 1; eIF4E, eukaryotic initiation factor 4E; eIF4B, eukaryotic initiation factor 4B; PDCD4, programmed cell death 4; eIF4A, eukaryotic initiation factor 4 A; SREBPs, sterol-regulatory element binding proteins; Lipin-1, lipid phosphate phosphohydrolase 1; PPARγ, peroxisome proliferator-activated receptor gamma; HIF1α, hypoxia-inducible factors; SGK, serum and glucocorticoid induced kinase; GSK3B, glycogen synthase kinase 3 β; ULK1, unc-51-like kinase 1; NADK, NAD kinase

BCAA and IGF-1 are critical regulators of muscle protein synthesis [67]. Insulin resistance is associated with dysregulated BCAA metabolism [68] and increased mTORC1 activity [69, 70]. Upon binding to their receptors, insulin and IGF-1 trigger phosphorylation of insulin receptor substrate 1 (IRS-1), which activates mTORC2 through the PI3K pathway. mTORC2 then stimulates mTORC1 signaling through AKT-mediated phosphorylation [52]. As displayed in Fig. 4, activated mTORC1 phosphorylates 4E-binding protein 1 (4E-BP1), releasing eukaryotic initiation factor 4E (eIF4E) to initiate translation and promote protein synthesis [57, 71]. Additionally, ribosomal protein S6 kinase 1 (S6K1) stimulates protein synthesis by activating eIF4B (a positive regulator of cap-dependent translation) while promoting the degradation of programmed cell death 4 (PDCD4) inhibitor eIF4A [57]. To prevent hyperactivation, S6K1 phosphorylates IRS-1 in a negative feedback loop, dampening the insulin-mediated PI3K-AKT pathway [72, 73].

Leucine is uniquely potent in stimulating mTORC1 signaling, with studies showing that Leu alone can activate mTORC1 as effectively as a complete AA mixture [74]. Ospina-Rojas et al. [75] found that supplementing broiler diets with Leu significantly increased the mRNA expression of *mTOR* and *S6K1* genes in muscle tissue, whereas supplementing with Val had no effect on the mRNA expression of these genes, highlighting Leu’s distinct role in mTOR pathway activation. Hao et al. [76] reported that Ile can stimulate milk protein and fat synthesis in mammary epithelial cells through the PI3K-BRG1-mTOR/SREBP-1c pathway in a dose-dependent manner. Similarly, Arriola Apelo et al. [77] demonstrated that Ile linearly increased mTOR and S6K1 phosphorylation in the mammary tissue of dairy cows, underscoring its role in lactation-related anabolic signaling.

Recent studies have uncovered novel mediators in mTOR signaling, revealing additional layers of complexity in its regulation. Jiang et al. [78] identified Wnt-1-induced secreted protein 3 (WISP3) as a potential new signaling factor linking mTOR with AA, making it a crucial regulator of AA-mediated milk protein synthesis and cell growth. Further expanding the regulatory landscape, Hayatine, a newly discovered mTOR-Rag A/C interaction inhibitor, was shown to suppress AA-induced mTORC1 activation [79], providing a potential therapeutic target for mTOR-related disorders. Luo et al. [80] found that Glycyl-tRNA synthase (GlyRS) is a key mediator in AA-induced mTOR expression and activation in bovine mammary epithelial cells, suggesting a direct connection between translational machinery and mTOR signaling. Beyond canonical pathways, Son et al. [81] demonstrated that downstream metabolites of Leu, such as acetyl-CoA, can independently activate mTORC1 by promoting EP-300-mediated acetylation of Raptor, bypassing traditional Leu-sensing mechanisms. As more key regulators of the mTOR signaling pathway are discovered, our understanding of this complex network will continue to expand, offering new insights into its role in metabolism, protein synthesis, and cell growth.

### Inhibition of protein degradation

**Fig. 5:**
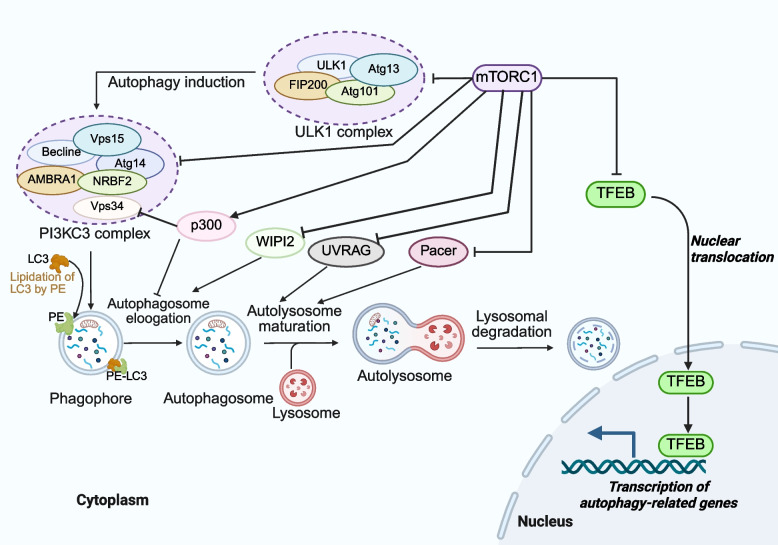
The mechanism of autophagy regulated by mTORC1. Autophagy initiation is coordinated by two kinases, ULK1 and PIK3C3. mTORC1 inhibits the activity of the ULK1 complex by phosphorylating ULK1 and Atg13. Additionally, mTORC1 suppresses the nucleation step of autophagy by phosphorylating Atg14, AMBRA1, and NRBF2 within the PI3KC3 complex. Furthermore, mTORC1 inhibits Vps34 activity and LC3 lipidation by phosphorylating p300 and WIPI2, thereby disrupting the recruitment of phosphatidylinositol phosphates and the LC3 conjugation system required for the autophagosome elongation. In addition, lipids play a crucial role in binding phosphatidylethanolamine (PE) to LC3, promoting the formation of autophagosome. Finally, mTORC1 negatively regulates the fusion of the autophagosomes with the lysosomes through the phosphorylation of UVRAG and Pacer. ULK1, unc-51-like kinase 1; ATG13, autophagy-related gene 13; TFEB, transcription factor EB; AMBRA1, autophagy and beclin 1 regulator 1

Protein deposition in skeletal muscle is a dynamic balance between protein synthesis and degradation, influenced by nutritional and hormonal cues [82]. The primary mechanisms of protein degradation include the ubiquitin–proteasome system (UPS), the autophagy-lysosomal system, calcium-activated proteases, and cysteine proteases [83, 84]. Among these, the UPS is the dominant pathway, accounting for over 80% of skeletal muscle protein degradation [85]. In this system, degradation begins with the activation of ubiquitin by the ubiquitin-activating enzyme E1 (UBE1), which forms a thioester bond between the C-terminal glycine of ubiquitin and the cysteine of E1. The activated ubiquitin is then transferred to the ubiquitin-conjugating enzyme E2 (UBE2) via a trans-thiolation reaction. Finally, ubiquitin ligase enzyme E3 (UBE3) facilitates the attachment of ubiquitin to the lysine residues of the target protein, marking them for proteasomal destruction [86]. The ubiquitinated protein is then recognized and processed by the proteasome, where it is broken down into short peptides or AA through protease catalyzed hydrolysis. Although the UPS is the primary degradation pathway, the autophagy-lysosomal system becomes critical during nutrient deprivation or metabolic stress. Importantly, mTORC1 suppresses both UPS and autophagy via downstream effectors (e.g., ULK1 and PIK3C3 complex), thereby shifting the balance toward net protein accretion and muscle preservation. As displayed in Fig. 5, BCAA-activated mTORC1 orchestrates a multi-tiered suppression of protein degradation pathways [36]. Specifically, mTORC1 suppresses autophagy by phosphorylating and inhibiting key autophagy regulators, such as unc-51 like autophagy activating kinase 1 (ULK-1) [87], autophagy related 13 Gene (ATG13) [88], transcription factor EB (TFEB) [89], and beclin-1-regulated autophagy (AMBRA1) [90]. mTORC1 prevents autophagosome formation by phosphorylating ATG13 and ULK1, thereby inhibiting their activity. Additionally, it blocks autophagosome maturation by phosphorylating autophagy related 14 (ATG14) [91]. TFEB, a key transcription factor, regulates the expression of genes essential for autophagy and lysosome biogenesis [92]. During nutrient deprivation or rapamycin-induced mTORC1 inhibition, dephosphorylated TFEB and related transcription factor E3 (TFE3) translocate to the nucleus to activate lysosomal and autophagy genes [57]. Conversely, nutrient-replete conditions promote mTORC1-mediated TFEB phosphorylation, sequestering it in the cytoplasm and suppressing autophagic flux [93]. This sophisticated regulatory network demonstrates how mTORC1 coordinately inhibits autophagy at multiple molecular levels, from initial vesicle formation to lysosomal gene expression.

mTORC1 exerts indirect control over the UPS through its regulation of AKT and FOXO signaling cascades. As the primary effector of PI3K-mediated insulin signaling, AKT mediates cellular proliferation and metabolic reprogramming via FOXO1/3a and NAD kinase (NADK) [94, 95]. The regulatory network extends to autophagy control through serum and glucocorticoid-regulated kinase (SGK), where mTORC2-SGK-1 inhibition potently induces autophagic flux in *Caenorhabditis elegans* [96]. AKT further coordinates metabolic responses by inactivating glycogen synthase kinase 3β (GSK3β) to support cell survival and glucose metabolism [57]. Crucially, AKT serves as a signaling nexus between mTOR complexes. It can phosphorylate tuberous sclerosis complex (TSC2) to activate mTORC1 while modifying mSin1 to modulate mTORC2 assembly and function [97, 98].

Under normal physiological conditions, skeletal muscle maintains proteostasis through balanced protein degradation and synthesis [99]. However, catabolic states such as fasting trigger accelerated proteolysis via the UPS and the autophagy-lysosomal system, leading to net muscle loss [100]. In response to diseases, inactivity, or acute endotoxemia, BCAA or Leu administration inhibits protein degradation through the UPS [101] and the autophagy-lysosomal system [102]. Zi et al. [103] revealed that mTORC1 phosphorylates serine/threonine kinase 11 interacting protein (STK11IP) to regulate lysosomal vacuolar-ATPase (V-ATPase) activity, providing a direct link between mTORC1 signaling and autophagy control. These findings establish BCAA as potent inhibitors of atrophy-induced proteolysis, suggesting their therapeutic potential for muscle preservation during energy deficit through dual modulation of major degradation pathways.

## Physiological functions of BCAA metabolites

### Leucine metabolite: HMB

Leu and its metabolites play pivotal roles in regulating protein metabolism, energy homeostasis, and cellular signaling. As the most metabolically active BCAA, approximately 80% of Leu is directly utilized for protein synthesis in pigs, while the remaining 20% undergoes conversion to α-KIC and β-hydroxy-β-methylbutyrate (HMB) in skeletal muscle [104]. Notably, endogenous HMB production represents only 0.66% of total Leu turnover [105]. In the liver mitochondria, about 90% of α-KIC is oxidized to isovaleryl-CoA (IVA-CoA), forming β-hydroxy**-**β-methylglutaryl-CoA (HMG-CoA), which is further metabolized to acetoacetate and acetyl-CoA. The remaining α-KIC is oxidized to HMB in the cytoplasm via α-KIC dioxygenase (KICD). HMB is cleared by the kidneys or metabolized into HMG-CoA, a precursor for cholesterol synthesis [106, 107].

As a leucine metabolite, HMB activates mTORC1 signaling via AKT phosphorylation to promote protein synthesis while suppressing degradation pathways [108, 109], though this occurs independently of IRS-1 [110]. Given its mechanistic overlap with Leu, HMB effectively substitutes for Leu in enhancing muscle protein synthesis [107, 111]. HMB concurrently reduces muscle catabolism and apoptosis by inhibiting the UPS and autophagy-lysosomal pathways [112, 113]. Cohen-Or et al. [114] demonstrated that HMB could induce myogenesis in C2C12 cells independently of AKT and mTOR, via phospholipase D2 (PLD2) activation of p70 ribosomal protein S6 kinase and ribosomal protein S6. Furthermore, HMB promotes muscle growth by stimulating the IGF-1 axis [115], enhancing AKT phosphorylation, and inhibiting autophagy.

Growing evidence from poultry and swine indicates that HMB modulates lipid metabolism and intestinal health in a dose and tissue-dependent manner. In broilers, dietary supplementation of approximately 0.10% HMB reduced hepatic fat deposition in parallel with shifts in gut microbial communities, suggesting involvement of the gut–liver axis in lipid regulation [116]. Furthermore, HMB alters muscle fatty acid composition in a muscle-specific manner. In leg muscle, 0.10%–0.15% HMB supplementation increased monounsaturated fatty acids (MUFA) while decreasing polyunsaturated fatty acids (PUFA), associated with the downregulation of lipogenic genes like *SREBP-1c* and reduced activities of lipid-metabolizing enzymes [117]. In breast muscle, 0.10% HMB exhibited an even stronger suppressive effect on lipogenesis, indicating distinct tissue-specific responses [117]. The benefits for intestinal integrity are supported by studies in weaned piglets, where HMB supplementation enhanced tight junction function, increased digestive enzyme activity, and modulated gut microbiota and short-chain fatty acid production, particularly under an LPS-induced inflammatory challenge [118]. Collectively, these findings demonstrate HMB’s potential to attenuate fat accumulation and maintain intestinal homeostasis.

### Valine metabolites: 3-HIB and BAIBA

The catabolism of Val generates 3-hydroxyisobutyrate (3-HIB) through the action of 3-hydroxyisobutyryl-CoA hydrolase on its precursor 3-hydroxyisobutyryl-CoA. This muscle-derived metabolite has emerged as a significant contributor to systemic insulin resistance through its effect on lipid metabolism [119]. 3-HIB stimulates fatty acid uptake in muscle tissue, leading to lipid accumulation and subsequent metabolic dysfunction. Jang et al. [120] indicated that 3-HIB could activate endothelial fatty acid transport, facilitating lipid uptake into muscle and promoting metabolic dysfunction in mice. Importantly, inhibition of 3-HIB synthesis has been shown to block peroxisome proliferators-activated receptor γ coactivator l α (PGC-1α) mediated endothelial fatty acid uptake. Recent studies further implicate HIBCH and 3-HIB in regulating lipid accumulation in the muscle and adipose tissues [121], with 3-HIB now recognized as a novel adipocyte-specific regulatory molecule. Its association with obesity, insulin resistance, and type 2 diabetes underscores the growing appreciation of BCAA metabolites as key modulators of meat quality. Che et al. [122] demonstrated that 3-HIB supplementation in late-gestation sows improved maternal-to-fetal fatty acid transport and neonatal oxidation, as evidenced by increased placental triglycerides and fatty acid transporters (SLC27A1 and FABP3), elevated piglet plasma MUFA and PUFA, and greater muscle CPT-1.

3-HIB is further metabolized to form methylmalonate semialdehyde, which interacts with 4-aminobutyrate aminotransferase to produce β-aminoisobutyric acid (BAIBA). This downstream metabolite exhibits significant metabolic benefits, particularly in enhancing muscle function through multiple mechanisms [123]. BAIBA exerts its effects primarily via the AMPK pathway. Jung et al. [124] found that moderate BAIBA levels not only increase AMPK phosphorylation but also reduce pro-inflammatory cytokines (TNFα and MCP-1) in LPS treated fibroblast cells, thereby mitigating inflammation associated insulin resistance. Beyond its anti-inflammatory properties, BAIBA improves lipid metabolism and induces white adipose tissue browning by upregulating the expression of peroxisome proliferator-activated receptor-α (PPARα) and uncoupling protein 1 (UCP-1) [125]. Minato et al. [126] further revealed that L-BAIBA treatment enhances AMPK and AKT phosphorylation, underscoring its beneficial effects on the liver, skeletal muscle, and adipose tissue. The beneficial effects of enhancing AKT and AMPK signaling are distinct yet complementary, with direct implications for body composition and muscle mass. However, their specific effects and mechanisms in livestock species require further investigation.

## Critical roles of BCAA in glucose and lipid metabolism

### Role of BCAA in glucose metabolism

The liver is a central metabolic organ that regulates energy homeostasis by coordinating the metabolism of skeletal muscle, adipose tissue, and other organs. BCAA regulate glucose and lipid metabolism primarily through the PI3K-AKT-mTOR pathway [10]. In skeletal muscle, elevated BCAA levels activate mTORC1/S6K1 signaling axis, which inhibits the PI3K/AKT pathway, thereby impairing insulin signaling and sensitivity [70, 127]. Experimental evidence supports this mechanism across diverse models. Leal Yepes et al. [128] reported that adding 550 g/d of rumen-protected BCAA into diets significantly increased plasma insulin levels in lactating cows. Conversely, Rivera et al. [129] found that excess BCAA reduces insulin sensitivity in cultured myotubes. Lynch and Adams [130] proposed an alternative perspective, arguing that increased BCAA may not directly induce insulin resistance but instead serve as a biomarker of underlying metabolic dysfunction.

Beyond direct signaling, BCAA metabolites also contribute to metabolic regulation. The metabolite BCKA has been implicated in insulin resistance [131]. Furthermore, impaired catabolism of BCAA and BCKA in the liver may disrupt systemic glucose homeostasis. This is demonstrated by liver-specific knockout of Ppm1k, a positive regulator of BCAA metabolism. Nishi et al. [132] found that Ppm1k knockout in mice impaired BCKA catabolism, leading to metabolite accumulation. This buildup suppressed the conversion of glycolytic precursors (pyruvate and lactate) into glucose via gluconeogenic pathways. While these findings suggest a potential mechanistic link between BCAA metabolism and insulin resistance, further research is needed to clarify whether BCAA directly drive insulin resistance or if their metabolites primarily contribute to metabolic dysregulation, as well as to disentangle their roles as causative agents versus biomarkers of impaired insulin action.

BCAA, particularly Leu, stimulate insulin secretion by allosterically activating glutamate dehydrogenase (GDH) [133]. In pancreatic β-cells, Leu uptake is mediated primarily by the LAT1 transporter, which is essential for Leu-stimulated insulin release. While Leu enhances insulin secretion, Ile and Val regulate glucose homeostasis through distinct mechanisms. Isabel et al. found that Ile and Val inhibit hepatic glucose production, though this regulatory effect diminishes following high-fat diet treatment [134]. A study in pigs fed low-protein diets demonstrated that supplementing BCAA increases uncoupling protein 3 (UCP3) expression in glycolytic skeletal muscle fibers, likely through activation of the AMPK-SIRT1-PGC-1α pathway [135]. Since UCP3 facilitates glucose transporter 4 (GLUT4) translocation, this mechanism links BCAA metabolism to improved glucose uptake in the skeletal muscle [135]. The metabolic impact of BCAA depends on their availability. On one hand, BCAA deficiency can disrupt glucose metabolism [136]. On the other hand, chronic BCAA accumulation downregulates the hexosamine biosynthetic pathway (HBP), leading to pyruvate dehydrogenase complex (PDH) inactivation and reduced glucose oxidation [137].

Additionally, mTORC1 enhances glycolysis by upregulating glycolytic enzymes via hypoxia-inducible factor 1α (HIF1α) activation [138, 139]. Conversely, AMPK plays a key role in maintaining systemic energy balance [140]. When cellular energy levels are low, AMPK is phosphorylated and activated by liver kinase B1 (LKB1) [141, 142]. Once activated, AMPK improves insulin sensitivity and promotes adipose tissue mobilization. Meanwhile, it also upregulates BCAA catabolic mechanisms in the skeletal muscle [143] and shifts metabolism toward increased catabolism and reduced anabolism by phosphorylating key proteins involved in mTORC1, lipid homeostasis, glycolysis, and mitochondrial function [144]. Collectively, these findings emphasize the significant role of BCAA and their metabolites in insulin sensitivity through its interactions with both mTORC1 and AMPK pathways. This understanding underscores the potential for targeted nutritional interventions to modulate these pathways for improved metabolic health in farm animals.

### Role of BCAA in lipid metabolism

BCAA catabolism influences lipid synthesis by contributing to the synthesis of N-acyl amino acids, branched-chain fatty acids, and odd-chain fatty acids [8]. Both white and brown adipocytes are functionally connected to circulating BCAA levels and systemic insulin resistance regulation [119]. Specifically, during adipogenesis, enzymes involved in BCAA catabolism increase, and BCAA oxidation supplies intermediates for cholesterol and odd-chain fatty acid synthesis. Elevated BCAA levels increase lipid accumulation during adipocyte differentiation [145]. Sterol-regulatory element binding proteins (SREBP) regulate lipogenesis [146], and BCAA activate mTORC1, which promotes SREBP1/2 nuclear translocation and processing via the S6K1-dependent mechanism, thereby stimulating lipogenesis. Additionally, mTORC1 phosphorylates Lipin-1, inhibiting its nuclear translocation and consequently relieving its suppression of SREBP-mediated lipogenic activity [147]. While these findings establish a clear connection between BCAA metabolism and lipid synthesis, it should be noted that current evidence primarily comes from studies on brown adipose tissue or in vitro models. Further research is needed to determine whether these mechanisms similarly operate in white adipose tissue of livestock.

While BCAA promote lipogenesis through mTORC1-SREBP signaling in adipocytes, emerging evidence reveals contrasting effects in other tissues and physiological contexts. Dietary BCAA supplementation paradoxically reduces lipid accumulation in the liver of largemouth bass (*Micropterus salmoides*) [148], suggesting tissue-specific regulatory mechanisms. This dichotomy extends to skeletal muscle, where Leu enhances fatty acid oxidation and mitochondrial biogenesis via AMPK and SIRT1 activation, as demonstrated in C2C12 myotubes in mice [149]. Similarly, dietary BCAA supplementation in laying hens suppressed de novo lipogenesis [150]. The isolated administration of Ile alone to obese mice further illustrates this complexity, showing selective reduction in white adipose tissue mass [151].

Emerging evidence demonstrates that BCAA deficiency substantially impacts systemic lipid metabolism. A Leu-deficient diet significantly suppresses hepatic lipogenesis, promotes lipolysis in white adipose tissue, and increases UCP1 expression in brown adipose tissue, collectively enhancing fat mobilization [152]. This phenomenon extends to other BCAA, as Ile or Val deficient diets similarly promote lipid catabolism while inhibiting hepatic lipid synthesis, reinforcing the crucial role of BCAA homeostasis in regulating adiposity [153]. These findings reveal a biphasic relationship between BCAA availability and lipid metabolism. While adequate BCAA levels support metabolic homeostasis, both deficiency and excess induce significant alterations in fat deposition and mobilization patterns. This dose-dependent response suggests an optimal range of BCAA intake for metabolic health. The regulation window likely varies by species, metabolic state, and individual BCAA composition, warranting further investigation to establish precise nutritional guidelines.

## Applications of dietary supplementation of BCAA on animal production

### Effects of BCAA supplementation on swine and poultry production

Dietary protein content is a key determinant of growth performance. Habibi et al. [154] reported that supplementing high levels of BCAA in protein-restricted diets partially mitigated the negative effects of protein restriction on growth performance in swine. Piglets fed a low-protein diet (LP, 17.1% crude protein) had lower serum BCAA levels, whereas piglets fed an LP + BCAA diet had BCAA concentrations comparable to those fed a normal-protein diet (20.9% crude protein). Supplementing BCAA in the LP diet improved growth performance, gut development, and intestinal AA transport protein expression in weaned piglets [155]. BCAA supplementation increased average daily gain (ADG) and feed intake in piglets, likely by modulating the hypothalamic general AA control or mTOR pathways, which could enhance feed intake and promote growth [156]. Tian et al. [157] demonstrated that BCAA deficiency may suppress feed intake by regulating AA T1R1/T1R3 receptors in the intestine or activating the hypothalamic general control nonderepressible 2 (GCN2)/eIF2α pathway. The intestinal T1R1/T1R3 receptors regulate food intake by modulating the release of gut hormones such as GLP-1, PYY, and CCK [158]. Specifically, BCAA deficiency upregulates intestinal T1R1/T3R3 expression, stimulating CCK secretion and subsequent feed intake reduction. At the central level, BCAA deficiency leads to accumulation of uncharged tRNAs that activate the GCN2 pathway, increasing eIF2α phosphorylation in the brain [159]. This hypothalamic eIF2α signaling is a well-established anorexigenic pathway [160], as evidenced by significantly elevated eIF2α phosphorylation in piglets fed BCAA-deficient diets.

Beyond appetite regulation, BCAA supplementation significantly impacts swine production. Zhang et al. [161] found that dietary supplementation of 0.4% Leu can prevent the reduction of loin eye area caused by low crude protein diets in fattening pigs. Luo et al. [162] reported that adding extra 35% of Ile improved pork quality by increasing intramuscular fat, reducing drip loss and shear force without affecting growth performance in crossbred barrows. Xu et al. [163] reported that the combination of low Val (SID Val = 0.31%) and high Ile (SID Ile = 0.53%) minimized drip loss. Conversely, a high Val diet is detrimental to meat quality, particularly reducing water holding capacity.

Balanced BCAA ratios can enhance growth and muscle synthesis while reducing lipid deposition in poultry. Wu [164] reported that the ideal BCAA profile in 10-day-old chicks follows a Leu:Val:Ile to Lys ratio of 1.13:0.68:0.58. Strategic reduction of crude protein with balanced BCAA supplementation has been shown to improve growth performance, meat tenderness, and carcass yield in broilers [165]. The anabolic effects of BCAA are primarily mediated through leucine's ability to stimulate muscle protein synthesis via *mTOR* and *S6K1* gene expressions in the muscle tissue signaling [166]. However, excessive Leu supplementation negatively affected overall growth performance, carcass weight, breast muscle weight, and body mineral content [167]. These negative effects stem from BCAA antagonism, where high Leu levels competitively inhibit the transport and functions of Val and Ile through shares AA transporters. This antagonism can be effectively counterbalanced by maintaining appropriate Val and Ile supplementation levels [168]. Ma et al. [169] found that dietary Ile supplementation improved growth performance and meat quality in broilers fed a low protein diet. Moreover, Ile reduced lipid deposition by activating the AMPK/mTOR and Leptin/JAK2/STAT3 pathways, which suppressed fat synthesis, promoted hepatic fat breakdown, and inhibited abdominal fat cell differentiation and proliferation [169]. Similarly, Jian et al. [150] found that high BCAA diets could inhibit de novo lipogenesis in laying hens by suppressing the tryptophan-ILA-AHR axis and MAPK9 signaling, while promoting fatty acid β-oxidation through PPAR-RXR activation and autophagy. These findings highlight the importance of maintaining precise BCAA levels in poultry nutrition to simultaneously optimize protein accretion and lipid metabolism while avoiding the detrimental effects of BCAA imbalance.

### Effects of BCAA supplementation on ruminant animal production

The effects of BCAA supplementation on ruminant growth and production performance varies across species and dietary conditions. Crucially, because free BCAA are quickly degraded in the rumen, dietary supplements must be rumen-protected to increase arterial BCAA concentrations and exert systemic effects. In goats, a low-protein diets (8.27% CP) reduced ADG, whereas supplementation with rumen-protected Leu significantly increased ADG [170, 171]. Similarly, supplementing 6 g/100 kg BW of Leu daily improved ADG in beef cattle, with altered plasma AA profiles, and increased free Leu bioavailability [3]. Dietary Leu supplementation also improved meat quality by reducing subcutaneous fat thickness and increasing marbling score and crude protein content in the *longissimus pectoral *muscle. However, supplementing 7.1 g/kg of Leu in 5-day-old lambs’ milk replacer had no effect on feed intake or ADG but lowered plasma urea nitrogen levels [172]. This suggests that young ruminants may have different Leu utilization patterns compared to growing or finishing animals.

In dairy cows, BCAA constitute approximately 40% of EAA supplied to the mammary glands [173]. Abomasal infusion of EAA without BCAA suppressed mTOR-mediated activation of eIF2Bε and eIF2α in the mammary glands, leading to decreased milk protein production in dairy cows [174]. However, studies suggest that BCAA supplementation may not directly enhance milk protein production when methionine and lysine are adequately supplied. Appuhamy et al. [175] found that, in the presence of adequate methionine and lysine, BCAA supplementation did not significantly enhance milk protein synthesis but reduced milk urea nitrogen levels, likely due to the activation of alternative protein synthesis pathways. Chen et al. [176] observed that adding 10.6 g/d rumen-protected Leu to diets significantly increased ADG without affecting milk yield in lactating goats. Similarly, Leal Yepes et al. [177] reported that postpartum supplementation with 550 g/d of BCAA only marginally increased milk yield and protein content, possibly because BCAA are preferentially metabolized for energy rather than protein synthesis in the mammary glands. In a parallel study, Leal Yepes et al. [128] demonstrated that supplementing 550 g/d of rumen-protected BCAA from calving to day 35 of lactation increased plasma insulin concentrations and reduced hepatic lipidosis. Overall, BCAA serve as critical substrates for transamination and nitrogen donation, supporting the de novo synthesis of non-EAA required for milk protein production [28].

Recent studies highlight the complex relationship between BCAA metabolism, residual feed intake (RFI), and metabolic disorders in cattle. Jorge-Smeding et al. [178] found that high-RFI cattle fed a high-starch diet exhibited BCAA accumulation, insulin resistance, and obesity, whereas these traits were not observed with high-fiber diets. In a follow-up work, Jorge-Smeding et al. [179] reported that elevated plasma BCAA in low-efficiency animals were associated with enhanced lipogenesis and fat deposition, possibly due to the ketogenic effects of Leu and Ile. Supporting these findings, a meta-analysis conducted by Duarte et al. [180] identified BCAA degradation as the sole metabolic pathway significantly correlated with RFI in beef cattle. These findings collectively position BCAA as key metabolic regulators of feed efficiency. Optimizing BCAA supplementation strategies could help mitigate metabolic inefficiencies and improve sustainability in ruminant production systems.

## Conclusion

BCAA serve primarily as substrates for protein synthesis. Beyond this role, they also function as key regulators in processes such as protein metabolism, energy homeostasis, and cellular signaling. Mechanistically, BCAA catabolism generates keto acids that enter the tricarboxylic acid cycle, directly supplying substrates for ATP production. Beyond energy provision, BCAA exert systemic metabolic control by regulating insulin secretion, glycogen synthesis, glycolysis, and fatty acid oxidation, thereby contributing to glucose-lipid equilibrium. At the molecular level, BCAA activate mTOR signaling pathways, driving cell proliferation and protein synthesis while suppressing autophagy, thus coordinating anabolic processes. These multifunctional properties establish BCAA as key theoretical targets for optimizing production efficiency, animal health, and product quality. Empirically, dietary BCAA supplementation consistently enhances growth performance and ADG, particularly in low-protein diets where their metabolic compensation effects are maximized. However, optimal BCAA supplementation levels vary significantly depending on animal species, developmental stage, and dietary nutrient composition. Ruminants often require rumen-protected techniques to bypass ruminal microbial degradation, whereas monogastric animals efficiently absorb BCAA post-intestinally. Despite these advancements, critical knowledge gaps remain, particularly regarding tissue-specific BCAA utilization kinetics and long-term effects of supplementation levels. Future research employing integrated omics approaches and dose–response trials will enable precision feeding strategies that fully and sustainably harness the potential of BCAA.

## Data Availability

Not applicable.

## Abbreviations

AA: Amino acid
3-HIB: 3-Hydroxyisobutyrate
AAT: Amino acid transporter
Ala: Alanine
AMPK: AMP-activated protein kinase
BAIBA: β-Aminoisobutyric acid
BCAA: Branched-chain amino acid
BCAT: Branched-chain amino acids transaminase
BCKA: Branched-chain keto acid
BCKDC: Branched-chain α-ketoacid dehydrogenase complex
EAA: Essential amino acid
Gln: Glutamic acid
Glu: Glutamine
HIF1α: Hypoxia inducible factor 1α
HMB: Beta-hydroxy-beta-methylbutyrate
IRS-1: Insulin receptor substrate 1
KIC: α-Ketoisocaproate acid
KIV: α-Ketoisovaleric acid
KMV: α-Ketonemethylvaleric acid
LAT1: L-type amino acid transporter 1
LKB1: Liver kinase B1
MCT: Monocarboxylate transporter
NADK: NAD kinase
PDH: Pyruvate dehydrogenase complex
PP2Cm: Protein phosphatase 2Cm
SNAT2: Sodium-coupled neutral amino acid transporter 2
SNAT3: Sodium-coupled neutral amino acid transporter 3
SNAT4: Sodium-coupled neutral amino acid transporter 4
TFEB: Transcription factor EB
TSC2: Tuberous sclerosis complex 2
α-KG: α-Ketoglutarate

## References

[1] Zumbaugh MD, Johnson SE, Shi TH, Gerrard DE. Molecular and biochemical regulation of skeletal muscle metabolism. J Anim Sci. 2022;100(8):skac035. 10.1093/jas/skac035. 35908794 10.1093/jas/skac035 PMC9339271

[2] Ferreira RP, Duarte JA. Protein turnover in skeletal muscle: looking at molecular regulation towards an active lifestyle. Int J Sports Med. 2023;44(11):763–77. 10.1055/a-2044-8277. 36854391 10.1055/a-2044-8277

[3] An J, Zhao X, Song Y, He H, Wang Z, Lan X, et al. High leucine bioavailability improves beef quality by altering serum metabolism in beef cattle. Meat Sci. 2024;220:109693. 10.1016/j.meatsci.2024.109693. 39486216 10.1016/j.meatsci.2024.109693

[4] Yi S, Ye B, Wang J, Yi X, Wang Y, Abudukelimu A, et al. Investigation of guanidino acetic acid and rumen-protected methionine induced improvements in longissimus lumborum muscle quality in beef cattle. Meat Sci. 2024;217:109624. 10.1016/j.meatsci.2024.109624. 39141966 10.1016/j.meatsci.2024.109624

[5] Shimomura Y, Murakami T, Nakai N, Nagasaki M, Harris RA. Exercise promotes BCAA catabolism: effects of BCAA supplementation on skeletal muscle during exercise. J Nutr. 2004;134(Suppl 6):1583s-s1587. 10.1093/jn/134.6.1583S. 15173434 10.1093/jn/134.6.1583S

[6] Moura A, Savageau MA, Alves R. Relative amino acid composition signatures of organisms and environments. PLoS ONE. 2013;8(10):e77319. 10.1371/journal.pone.0077319. 24204807 10.1371/journal.pone.0077319 PMC3808408

[7] Mackle TR, Dwyer DA, Bauman DE. Effects of branched-chain amino acids and sodium caseinate on milk protein concentration and yield from dairy cows. J Dairy Sci. 1999;82(1):161–71. 10.3168/jds.S0022-0302(99)75220-3. 10022018 10.3168/jds.S0022-0302(99)75220-3

[8] Neinast M, Murashige D, Arany Z. Branched chain amino acids. Annu Rev Physiol. 2019;81:139–64. 10.1146/annurev-physiol-020518-114455. 30485760 10.1146/annurev-physiol-020518-114455 PMC6536377

[9] Suryawan A, Nguyen HV, Almonaci RD, Davis TA. Differential regulation of protein synthesis in skeletal muscle and liver of neonatal pigs by leucine through an mTORC1-dependent pathway. J Anim Sci Biotechnol. 2012;3:3. 10.1186/2049-1891-3-3. 10.1186/2049-1891-3-3 PMC3366465 22675606

[10] Nie C, He T, Zhang W, Zhang G, Ma X. Branched chain amino acids: beyond nutrition metabolism. Int J Mol Sci. 2018;19(4):954. 10.3390/ijms19040954. 29570613 10.3390/ijms19040954 PMC5979320

[11] Chand D, Varma SD, Kushwaha RPS. Active transport of glycine by rumen epithelium of betal goats. J Dairy Sci. 1968;51(9):1420–2. 10.3168/jds.S0022-0302(68)87205-4. 5673682 10.3168/jds.S0022-0302(68)87205-4

[12] Zhang J, Xu Y, Li D, Fu L, Zhang X, Bao Y, et al. Review of the correlation of LAT1 with diseases: mechanism and treatment. Front Chem. 2020;8:564809. 10.3389/fchem.2020.564809. 33195053 10.3389/fchem.2020.564809 PMC7606929

[13] Verrey F, Closs EI, Wagner CA, Palacin M, Endou H, Kanai Y. CATs and HATs: the SLC7 family of amino acid transporters. Pflugers Arch. 2004;447(5):532–42. 10.1007/s00424-003-1086-z. 14770310 10.1007/s00424-003-1086-z

[14] Kahlhofer J, Teis D. The human LAT1-4F2hc (SLC7A5-SLC3A2) transporter complex: physiological and pathophysiological implications. Basic Clin Pharmacol Toxicol. 2023;133(5):459–72. 10.1111/bcpt.13821. 36460306 10.1111/bcpt.13821 PMC11497297

[15] Kandasamy P, Gyimesi G, Kanai Y, Hediger MA. Amino acid transporters revisited: new views in health and disease. Trends Biochem Sci. 2018;43(10):752–89. 10.1016/j.tibs.2018.05.003. 30177408 10.1016/j.tibs.2018.05.003

[16] Hutson SM, Sweatt AJ, Lanoue KF. Branched-chain amino acid metabolism: implications for establishing safe intakes. J Nutr. 2005;135(Suppl 6):1557s-s1564. 10.1093/jn/135.6.1557S. 15930469 10.1093/jn/135.6.1557S

[17] White PJ, McGarrah RW, Herman MA, Bain JR, Shah SH, Newgard CB. Insulin action, type 2 diabetes, and branched-chain amino acids: a two-way street. Mol Metab. 2021;52:101261. 10.1016/j.molmet.2021.101261. 34044180 10.1016/j.molmet.2021.101261 PMC8513145

[18] Hou X, Song S, Xu Z, Shi Y, Yang Y, Zhang L, et al. Prolactin upregulates amino acid uptake in dairy cow mammary epithelial cells via LAT1. J Dairy Sci. 2024;107(11):9948–59. 10.3168/jds.2024-24746. 38945262 10.3168/jds.2024-24746

[19] Mansoori S, Ho MY, Ng KK, Cheng KK. Branched-chain amino acid metabolism: pathophysiological mechanism and therapeutic intervention in metabolic diseases. Obes Rev. 2025;26(2):e13856. 10.1111/obr.13856. 39455059 10.1111/obr.13856 PMC11711082

[20] Wang Q, Holst J. L-type amino acid transport and cancer: targeting the mTORC1 pathway to inhibit neoplasia. Am J Cancer Res. 2015;5(4):1281–94. 26101697 PMC4473310

[21] Zhao X, Sakamoto S, Wei J, Pae S, Saito S, Sazuka T, et al. Contribution of the L-type amino acid transporter family in the diagnosis and treatment of prostate cancer. Int J Mol Sci. 2023;24(7):6178. 10.3390/ijms24076178. 37047148 10.3390/ijms24076178 PMC10094571

[22] Yoneshiro T, Wang Q, Tajima K, Matsushita M, Maki H, Igarashi K, et al. BCAA catabolism in brown fat controls energy homeostasis through SLC25A44. Nature. 2019;572(7771):614–9. 10.1038/s41586-019-1503-x. 31435015 10.1038/s41586-019-1503-x PMC6715529

[23] Ramírez de la Piscina-Viúdez X, Álvarez-Herms J, Bonilla DA, Castañeda-Babarro A, Larruskain J, Díaz-Ramírez J, et al. Putative role of MCT1 rs1049434 polymorphism in high-intensity endurance performance: concept and basis to understand possible individualization stimulus. Sports, 2021;9(10):143. 10.3390/sports9100143. 10.3390/sports9100143 PMC8537363 34678924

[24] Hewton KG, Johal AS, Parker SJ. Transporters at the interface between cytosolic and mitochondrial amino acid metabolism. Metabolites. 2021;11(2):112. 10.3390/metabo11020112. 33669382 10.3390/metabo11020112 PMC7920303

[25] Kanai Y, Clémençon B, Simonin A, Leuenberger M, Lochner M, Weisstanner M, et al. The SLC1 high-affinity glutamate and neutral amino acid transporter family. Mol Aspects Med. 2013;34(2–3):108–20. 10.1016/j.mam.2013.01.001. 23506861 10.1016/j.mam.2013.01.001

[26] Kondou H, Kawai M, Tachikawa K, Kimoto A, Yamagata M, Koinuma T, et al. Sodium-coupled neutral amino acid transporter 4 functions as a regulator of protein synthesis during liver development. Hepatol Res. 2013;43(11):1211–23. 10.1111/hepr.12069. 23607685 10.1111/hepr.12069

[27] Bröer S. Amino acid transporters as modulators of glucose homeostasis. Trends Endocrinol Metab. 2022;33(2):120–35. 10.1016/j.tem.2021.11.004. 34924221 10.1016/j.tem.2021.11.004

[28] Lapierre H, Berthiaume R, Raggio G, Thivierge MC, Doepel L, Pacheco D, et al. The route of absorbed nitrogen into milk protein. Anim Sci. 2005;80(1):11–22. 10.1079/ASC41330011.

[29] Clark JH, Klusmeyer TH, Cameron MR. Microbial protein synthesis and flows of nitrogen fractions to the duodenum of dairy cows. J Dairy Sci. 1992;75(8):2304–23. 10.3168/jds.S0022-0302(92)77992-2. 1401380 10.3168/jds.S0022-0302(92)77992-2

[30] Estes KA, White RR, Yoder PS, Pilonero T, Schramm H, Lapierre H, et al. An in vivo stable isotope–based approach for assessment of absorbed amino acids from individual feed ingredients within complete diets. J Dairy Sci. 2018;101(8):7040–60. 10.3168/jds.2017-13447. 29778479 10.3168/jds.2017-13447

[31] Lobley GE, Shen X, Le G, Bremner DM, Milne E, Calder AG, et al. Oxidation of essential amino acids by the ovine gastrointestinal tract. Br J Nutr. 2003;89(5):617–30. 10.1079/bjn2003831. 12720582 10.1079/BJN2003831

[32] Lobley GE. Control of the metabolic fate of amino acids in ruminants: a review. J Anim Sci. 1992;70(10):3264–75. 10.2527/1992.70103264x. 1429303 10.2527/1992.70103264x

[33] Kristensen NB, Guoyao W. Metabolic functions of the porcine liver. In: Knudsen KEB, Kjeldsen NJ, Poulsen HD, Jensen BB, editors. Nutritional physiology of pigs - Online publication. Foulum: Videncenter for Svineproduktion; 2012.

[34] Harper AE, Miller RH, Block KP. Branched-chain amino acid metabolism. Annu Rev Nutr. 1984;4:409–54. 10.1146/annurev.nu.04.070184.002205. 6380539 10.1146/annurev.nu.04.070184.002205

[35] Shimomura Y, Kitaura Y. Physiological and pathological roles of branched-chain amino acids in the regulation of protein and energy metabolism and neurological functions. Pharmacol Res. 2018;133:215–7. 10.1016/j.phrs.2018.05.014. 29803540 10.1016/j.phrs.2018.05.014

[36] Mann G, Mora S, Madu G, Adegoke OAJ. Branched-chain amino acids: catabolism in skeletal muscle and implications for muscle and whole-body metabolism. Front Physiol. 2021;12:702826. 10.3389/fphys.2021.702826. 34354601 10.3389/fphys.2021.702826 PMC8329528

[37] Chen L, Chen Z, Zheng P, Sun J, Zeng AP. Study and reengineering of the binding sites and allosteric regulation of biosynthetic threonine deaminase by isoleucine and valine in *Escherichia coli*. Appl Microbiol Biotechnol. 2013;97(7):2939–49. 10.1007/s00253-012-4176-z. 22669632 10.1007/s00253-012-4176-z

[38] Suryawan A, Hawes JW, Harris RA, Shimomura Y, Jenkins AE, Hutson SM. A molecular model of human branched-chain amino acid metabolism. Am J Clin Nutr. 1998;68(1):72–81. 10.1093/ajcn/68.1.72. 9665099 10.1093/ajcn/68.1.72

[39] Biswas D, Duffley L, Pulinilkunnil T. Role of branched-chain amino acid–catabolizing enzymes in intertissue signaling, metabolic remodeling, and energy homeostasis. FASEB J. 2019;33(8):8711–31. 10.1096/fj.201802842RR. 31084571 10.1096/fj.201802842RR

[40] Adeva-Andany MM, López-Maside L, Donapetry-García C, Fernández-Fernández C, Sixto-Leal C. Enzymes involved in branched-chain amino acid metabolism in humans. Amino Acids. 2017;49(6):1005–28. 10.1007/s00726-017-2412-7. 28324172 10.1007/s00726-017-2412-7

[41] Bonvini A, Coqueiro AY, Tirapegui J, Calder PC, Rogero MM. Immunomodulatory role of branched-chain amino acids. Nutr Rev. 2018;76(11):840–56. 10.1093/nutrit/nuy037. 30124936 10.1093/nutrit/nuy037

[42] Dimou A, Tsimihodimos V, Bairaktari E. The critical role of the branched chain amino acids (BCAAs) catabolism-regulating enzymes, branched-chain aminotransferase (BCAT) and branched-chain α-keto acid dehydrogenase (BCKD), in human pathophysiology. Int J Mol Sci. 2022;23(7):4022. 10.3390/ijms23074022. 35409380 10.3390/ijms23074022 PMC8999875

[43] Neinast MD, Jang C, Hui S, Murashige DS, Chu Q, Morscher RJ, et al. Quantitative analysis of the whole-body metabolic fate of branched-chain amino acids. Cell Metab. 2019;29(2):417-29.e4. 10.1016/j.cmet.2018.10.013. 30449684 10.1016/j.cmet.2018.10.013 PMC6365191

[44] Blair MC, Neinast MD, Arany Z. Whole-body metabolic fate of branched-chain amino acids. Biochem J. 2021;478(4):765–76. 10.1042/bcj20200686. 33626142 10.1042/BCJ20200686 PMC9183206

[45] Jewell JL, Kim YC, Russell RC, Yu FX, Park HW, Plouffe SW, et al. Differential regulation of mTORC1 by leucine and glutamine. Science. 2015;347(6218):194–8. 10.1126/science.1259472. 25567907 10.1126/science.1259472 PMC4384888

[46] Shimomura Y, Obayashi M, Murakami T, Harris RA. Regulation of branched-chain amino acid catabolism: nutritional and hormonal regulation of activity and expression of the branched-chain alpha-keto acid dehydrogenase kinase. Curr Opin Clin Nutr Metab Care. 2001;4(5):419–23. 10.1097/00075197-200109000-00013. 11568504 10.1097/00075197-200109000-00013

[47] White PJ, McGarrah RW, Grimsrud PA, Tso S-C, Yang W-H, Haldeman JM, et al. The BCKDH kinase and phosphatase integrate BCAA and lipid metabolism via regulation of ATP-citrate lyase. Cell Metab. 2018;27(6):1281-93.e7. 10.1016/j.cmet.2018.04.015. 29779826 10.1016/j.cmet.2018.04.015 PMC5990471

[48] Wynn RM, Kato M, Machius M, Chuang JL, Li J, Tomchick DR, et al. Molecular mechanism for regulation of the human mitochondrial branched-chain α-ketoacid dehydrogenase complex by phosphorylation. Structure. 2004;12(12):2185–96. 10.1016/j.str.2004.09.013. 15576032 10.1016/j.str.2004.09.013

[49] Arp NL, Seim GL, Votava JA, Josephson J, Fan J. Reactive nitrogen species inhibit branched chain alpha-ketoacid dehydrogenase complex and impact muscle cell metabolism. J Biol Chem. 2023;299(11):105333. 10.1016/j.jbc.2023.105333. 37827290 10.1016/j.jbc.2023.105333 PMC10656228

[50] Manoli I, Venditti CP. Disorders of branched chain amino acid metabolism. Transl Sci Rare Dis. 2016;1(2):91–110. 10.3233/trd-160009. 29152456 10.3233/TRD-160009 PMC5685199

[51] Tanianskii DA, Jarzebska N, Birkenfeld AL, O’Sullivan JF, Rodionov RN. Beta-aminoisobutyric acid as a novel regulator of carbohydrate and lipid metabolism. Nutrients. 2019;11(3):524. 10.3390/nu11030524. 30823446 10.3390/nu11030524 PMC6470580

[52] Goul C, Peruzzo R, Zoncu R. The molecular basis of nutrient sensing and signalling by mTORC1 in metabolism regulation and disease. Nat Rev Mol Cell Biol. 2023;24(12):857–75. 10.1038/s41580-023-00641-8. 37612414 10.1038/s41580-023-00641-8

[53] Saxton RA, Sabatini DM. mTOR signaling in growth, metabolism, and disease. Cell. 2017;168(6):960–76. 10.1016/j.cell.2017.02.004. 28283069 10.1016/j.cell.2017.02.004 PMC5394987

[54] Bar-Peled L, Chantranupong L, Cherniack AD, Chen WW, Ottina KA, Grabiner BC, et al. A tumor suppressor complex with GAP activity for the Rag GTPases that signal amino acid sufficiency to mTORC1. Science. 2013;340(6136):1100–6. 23723238 10.1126/science.1232044 PMC3728654

[55] Wolfson RL, Chantranupong L, Saxton RA, Shen K, Scaria SM, Cantor JR, et al. Sestrin2 is a leucine sensor for the mTORC1 pathway. Science. 2016;351(6268):43–8. 10.1126/science.aab2674. 26449471 10.1126/science.aab2674 PMC4698017

[56] Kanzaki K, Wada M. Effects of leucine ingestion and contraction on the Sestrin/GATOR2 pathway and mTORC1 activation in rat fast-twitch muscle. J Nutr. 2023;153(8):2228–36. 10.1016/j.tjnut.2023.06.011. 37328110 10.1016/j.tjnut.2023.06.011

[57] Liu GY, Sabatini DM. mTOR at the nexus of nutrition, growth, ageing and disease. Nat Rev Mol Cell Biol. 2020;21(4):183–203. 10.1038/s41580-019-0199-y. 31937935 10.1038/s41580-019-0199-y PMC7102936

[58] Khalil MI, Ali MM, Holail J, Houssein M. Growth or death? Control of cell destiny by mTOR and autophagy pathways. Prog Biophys Mol Biol. 2023;185:39–55. 10.1016/j.pbiomolbio.2023.10.002. 37944568 10.1016/j.pbiomolbio.2023.10.002

[59] Peng M, Yin N, Li MO. SZT2 dictates GATOR control of mTORC1 signalling. Nature. 2017;543(7645):433–7. 10.1038/nature21378. 28199315 10.1038/nature21378 PMC5570594

[60] Wolfson RL, Chantranupong L, Wyant GA, Gu X, Orozco JM, Shen K, et al. KICSTOR recruits GATOR1 to the lysosome and is necessary for nutrients to regulate mTORC1. Nature. 2017;543(7645):438–42. 10.1038/nature21423. 28199306 10.1038/nature21423 PMC5360989

[61] Patti ME, Brambilla E, Luzi L, Landaker EJ, Kahn CR. Bidirectional modulation of insulin action by amino acids. J Clin Invest. 1998;101(7):1519–29. 10.1172/jci1326. 9525995 10.1172/JCI1326 PMC508730

[62] Deng L, Chen L, Zhao L, Xu Y, Peng X, Wang X, et al. Ubiquitination of Rheb governs growth factor-induced mTORC1 activation. Cell Res. 2019;29(2):136–50. 10.1038/s41422-018-0120-9. 30514904 10.1038/s41422-018-0120-9 PMC6355928

[63] Cheng Q, Beltran VD, Chan SM, Brown JR, Bevington A, Herbert TP. System-L amino acid transporters play a key role in pancreatic β-cell signalling and function. J Mol Endocrinol. 2016;56(3):175–87. 10.1530/jme-15-0212. 26647387 10.1530/JME-15-0212

[64] Zhang T, Wang R, Wang Z, Wang X, Wang F, Ding J. Structural basis for ragulator functioning as a scaffold in membrane-anchoring of Rag GTPases and mTORC1. Nat Commun. 2017;8:1394. 10.1038/s41467-017-01567-4. 10.1038/s41467-017-01567-4 PMC5680233 29123114

[65] Gollwitzer P, Grützmacher N, Wilhelm S, Kümmel D, Demetriades C. A Rag GTPase dimer code defines the regulation of mTORC1 by amino acids. Nat Cell Biol. 2022;24(9):1394–406. 10.1038/s41556-022-00976-y. 36097072 10.1038/s41556-022-00976-y PMC9481461

[66] Linde-Garelli KY, Rogala KB. Structural mechanisms of the mTOR pathway. Curr Opin Struct Biol. 2023;82:102663. 10.1016/j.sbi.2023.102663. 37572585 10.1016/j.sbi.2023.102663

[67] Saeki C, Kanai T, Nakano M, Oikawa T, Torisu Y, Saruta M, et al. Low serum branched-chain amino acid and insulin-like growth factor-1 levels are associated with sarcopenia and slow gait speed in patients with liver cirrhosis. J Clin Med. 2020;9(10):3239. 10.3390/jcm9103239. 33050430 10.3390/jcm9103239 PMC7600046

[68] Wang J, Liu Y, Lian K, Shentu X, Fang J, Shao J, et al. BCAA catabolic defect alters glucose metabolism in lean mice. Front Physiol. 2019;10:1140. 10.3389/fphys.2019.01140. 31551816 10.3389/fphys.2019.01140 PMC6738029

[69] Moghei M, Tavajohi-Fini P, Beatty B, Adegoke OA. Ketoisocaproic acid, a metabolite of leucine, suppresses insulin-stimulated glucose transport in skeletal muscle cells in a BCAT2-dependent manner. Am J Physiol Cell Physiol. 2016;311(3):C518–27. 10.1152/ajpcell.00062.2016. 27488662 10.1152/ajpcell.00062.2016 PMC5129764

[70] Yoon MS. The emerging role of branched-chain amino acids in insulin resistance and metabolism. Nutrients. 2016;8(7):405. 10.3390/nu8070405. 27376324 10.3390/nu8070405 PMC4963881

[71] Levitt DE, Luk HY, Vingren JL. Alcohol, resistance exercise, and mTOR pathway signaling: an evidence-based narrative review. Biomolecules. 2022;13(1):2. 10.3390/biom13010002. 36671386 10.3390/biom13010002 PMC9855961

[72] Harrington LS, Findlay GM, Gray A, Tolkacheva T, Wigfield S, Rebholz H, et al. The TSC1-2 tumor suppressor controls insulin-PI3K signaling via regulation of IRS proteins. J Cell Biol. 2004;166(2):213–23. 10.1083/jcb.200403069. 15249583 10.1083/jcb.200403069 PMC2172316

[73] Shah OJ, Wang Z, Hunter T. Inappropriate activation of the TSC/Rheb/mTOR/S6K cassette induces IRS1/2 depletion, insulin resistance, and cell survival deficiencies. Curr Biol. 2004;14(18):1650–6. 10.1016/j.cub.2004.08.026. 15380067 10.1016/j.cub.2004.08.026

[74] Dodd KM, Tee AR. Leucine and mTORC1: a complex relationship. Am J Physiol Endocrinol Metab. 2012;302(11):E1329–42. 10.1152/ajpendo.00525.2011. 22354780 10.1152/ajpendo.00525.2011

[75] Ospina-Rojas IC, Murakami AE, do Amaral Duarte CR, Pozza PC, Rossi RM, Gasparino E. Performance, diameter of muscle fibers, and gene expression of mechanistic target of rapamycin in pectoralis major muscle of broilers supplemented with leucine and valine. Can J Anim Sci, 2018;99(1):168–78. 10.1139/cjas-2018-0020.

[76] Hao Q, Wang Z, Wang L, Han M, Zhang M, Gao X. Isoleucine stimulates mTOR and SREBP-1c gene expression for milk synthesis in mammary epithelial cells through BRG1-mediated chromatin remodelling. Brit J Nutr. 2023;129(4):553–63. 10.1017/S0007114522001544. 10.1017/S0007114522001544 35593529

[77] Arriola Apelo SI, Singer LM, Lin XY, McGilliard ML, St-Pierre NR, Hanigan MD. Isoleucine, leucine, methionine, and threonine effects on mammalian target of rapamycin signaling in mammary tissue. J Dairy Sci. 2014;97(2):1047–56. 10.3168/jds.2013-7348. 24359813 10.3168/jds.2013-7348

[78] Jiang N, Wang Y, Yu Z, Hu L, Liu C, Gao X, et al. WISP3 (CCN6) regulates milk protein synthesis and cell growth through mTOR signaling in dairy cow mammary epithelial cells. DNA Cell Biol. 2015;34(8):524–33. 10.1089/dna.2015.2829. 26061139 10.1089/dna.2015.2829

[79] Lu M, Yu L, Yang Y, Zhu J, Qiang S, Wang X, et al. Hayatine inhibits amino acid-induced mTORC1 activation as a novel mTOR-Rag A/C interaction disruptor. Biochem Bioph Res Co. 2021;583:71–8. 10.1016/j.bbrc.2021.10.014. 10.1016/j.bbrc.2021.10.014 34735882

[80] Luo C, Qi H, Huang X, Li M, Zhang L, Lin Y, et al. GlyRS is a new mediator of amino acid-induced milk synthesis in bovine mammary epithelial cells. J Cell Physiol. 2019;234(3):2973–83. 10.1002/jcp.27115. 30171693 10.1002/jcp.27115

[81] Son SM, Park SJ, Lee H, Siddiqi F, Lee JE, Menzies FM, et al. Leucine signals to mTORC1 via Its metabolite acetyl-coenzyme A. Cell Metab. 2019;29(1):192-201.e7. 10.1016/j.cmet.2018.08.013. 30197302 10.1016/j.cmet.2018.08.013 PMC6331339

[82] Sandri M. Signaling in muscle atrophy and hypertrophy. Physiology (Bethesda). 2008;23:160–70. 10.1152/physiol.00041.2007. 18556469 10.1152/physiol.00041.2007

[83] Anthony TG. Mechanisms of protein balance in skeletal muscle. Domest Anim Endocrinol. 2016;Suppl 56:S23–32. 10.1016/j.domaniend.2016.02.012. 10.1016/j.domaniend.2016.02.012 PMC4926040 27345321

[84] Bell RA, Al-Khalaf M, Megeney LA. The beneficial role of proteolysis in skeletal muscle growth and stress adaptation. Skelet Muscle. 2016;6:19. 10.1186/s13395-016-0086-6. 10.1186/s13395-016-0086-6 PMC4822268 27054028

[85] Wing SS, Lecker SH, Jagoe RT. Proteolysis in illness-associated skeletal muscle atrophy: from pathways to networks. Crit Rev Clin Lab Sci. 2011;48(2):49–70. 10.3109/10408363.2011.586171. 21699435 10.3109/10408363.2011.586171 PMC5734931

[86] Atta H, Kassem DH, Kamal MM, Hamdy NM. Targeting the ubiquitin proteasome system in cancer stem cells. Trends Cell Biol. 2024;35(2):97–101. 10.1016/j.tcb.2024.11.011. 39721924 10.1016/j.tcb.2024.11.011

[87] Hosokawa N, Hara T, Kaizuka T, Kishi C, Takamura A, Miura Y, et al. Nutrient-dependent mTORC1 association with the ULK1-Atg13-FIP200 complex required for autophagy. Mol Biol Cell. 2009;20(7):1981–91. 10.1091/mbc.e08-12-1248. 19211835 10.1091/mbc.E08-12-1248 PMC2663915

[88] Foerster EG, Mukherjee T, Cabral-Fernandes L, Rocha JDB, Girardin SE, Philpott DJ. How autophagy controls the intestinal epithelial barrier. Autophagy. 2022;18(1):86–103. 10.1080/15548627.2021.1909406. 33906557 10.1080/15548627.2021.1909406 PMC8865220

[89] Martina JA, Chen Y, Gucek M, Puertollano R. mTORC1 functions as a transcriptional regulator of autophagy by preventing nuclear transport of TFEB. Autophagy. 2012;8(6):903–14. 10.4161/auto.19653. 22576015 10.4161/auto.19653 PMC3427256

[90] Nazio F, Strappazzon F, Antonioli M, Bielli P, Cianfanelli V, Bordi M, et al. mTOR inhibits autophagy by controlling ULK1 ubiquitylation, self-association and function through AMBRA1 and TRAF6. Nat Cell Biol. 2013;15(4):406–16. 10.1038/ncb2708. 23524951 10.1038/ncb2708

[91] Odle RI, Walker SA, Oxley D, Kidger AM, Balmanno K, Gilley R, et al. An mTORC1-to-CDK1 switch maintains autophagy suppression during mitosis. Mol Cell. 2020;77(2):228-40.e7. 10.1016/j.molcel.2019.10.016. 31733992 10.1016/j.molcel.2019.10.016 PMC6964153

[92] Fang Z, Xu Y, Liu G, Shao Q, Niu X, Tai W, et al. Narirutin activates TFEB (transcription factor EB) to protect against acetaminophen-induced liver injury by targeting PPP3/calcineurin. Autophagy. 2023;19(8):2240–56. 10.1080/15548627.2023.2179781. 36779633 10.1080/15548627.2023.2179781 PMC10351474

[93] Alesi N, Khabibullin D, Rosenthal DM, Akl EW, Cory PM, Alchoueiry M, et al. TFEB drives mTORC1 hyperactivation and kidney disease in tuberous sclerosis complex. Nat Commun. 2024;15:406. 10.1038/s41467-023-44229-4. 10.1038/s41467-023-44229-4 PMC10776564 38195686

[94] Webb AE, Brunet A. FOXO transcription factors: key regulators of cellular quality control. Trends Biochem Sci. 2014;39(4):159–69. 10.1016/j.tibs.2014.02.003. 24630600 10.1016/j.tibs.2014.02.003 PMC4021867

[95] Hoxhaj G, Ben-Sahra I, Lockwood SE, Timson RC, Byles V, Henning GT, et al. Direct stimulation of NADP^+^ synthesis through Akt-mediated phosphorylation of NAD kinase. Science. 2019;363(6431):1088–92. 10.1126/science.aau3903. 30846598 10.1126/science.aau3903 PMC7261235

[96] Aspernig H, Heimbucher T, Qi W, Gangurde D, Curic S, Yan Y, et al. Mitochondrial perturbations couple mTORC2 to autophagy in *C. elegans*. Cell Rep, 2019;29(6):1399–409.e5. 10.1016/j.celrep.2019.09.072. 10.1016/j.celrep.2019.09.072 31693882

[97] Humphrey SJ, Yang G, Yang P, Fazakerley DJ, Stöckli J, Yang JY, et al. Dynamic adipocyte phosphoproteome reveals that Akt directly regulates mTORC2. Cell Metab. 2013;17(6):1009–20. 10.1016/j.cmet.2013.04.010. 23684622 10.1016/j.cmet.2013.04.010 PMC3690479

[98] Jia R, Bonifacino JS. Lysosome positioning influences mTORC2 and AKT signaling. Mol Cell. 2019;75(1):26-38.e3. 10.1016/j.molcel.2019.05.009. 31130364 10.1016/j.molcel.2019.05.009 PMC7446307

[99] Sandri M. New findings of lysosomal proteolysis in skeletal muscle. Curr Opin Clin Nutr Metab Care. 2011;14(3):223–9. 10.1097/MCO.0b013e3283457a75. 21415731 10.1097/MCO.0b013e3283457a75

[100] Thivierge MC, Bush JA, Suryawan A, Nguyen HV, Orellana RA, Burrin DG, et al. Positive net movements of amino acids in the hindlimb after overnight food deprivation contribute to sustaining the elevated anabolism of neonatal pigs. J Appl Physiol. 2008;105(6):1959–66. 10.1152/japplphysiol.90352.2008. 18801965 10.1152/japplphysiol.90352.2008 PMC2612466

[101] Hernandez-García AD, Columbus DA, Manjarín R, Nguyen HV, Suryawan A, Orellana RA, et al. Leucine supplementation stimulates protein synthesis and reduces degradation signal activation in muscle of newborn pigs during acute endotoxemia. Am J Physiol Endocrinol Metab. 2016;311(4):E791-e801. 10.1152/ajpendo.00217.2016. 27624100 10.1152/ajpendo.00217.2016 PMC5241557

[102] Boutry C, El-Kadi SW, Suryawan A, Wheatley SM, Orellana RA, Kimball SR, et al. Leucine pulses enhance skeletal muscle protein synthesis during continuous feeding in neonatal pigs. Am J Physiol Endocrinol Metab. 2013;305(5):E620–31. 10.1152/ajpendo.00135.2013. 23839523 10.1152/ajpendo.00135.2013 PMC3761169

[103] Zi Z, Zhang Z, Feng Q, Kim C, Wang X-D, Scherer PE, et al. Quantitative phosphoproteomic analyses identify STK11IP as a lysosome-specific substrate of mTORC1 that regulates lysosomal acidification. Nat Commun. 2022;13:1760. 10.1038/s41467-022-29461-8. 10.1038/s41467-022-29461-8 PMC8976005 35365663

[104] Duan Y, Li F, Li Y, Tang Y, Kong X, Feng Z, et al. The role of leucine and its metabolites in protein and energy metabolism. Amino Acids. 2016;48(1):41–51. 10.1007/s00726-015-2067-1. 26255285 10.1007/s00726-015-2067-1

[105] Walker DK, Thaden JJ, Wierzchowska-McNew A, Engelen M, Deutz NEP. Determination of β-hydroxy-β-methylbutyrate concentration and enrichment in human plasma using chemical ionization gas chromatography tandem mass spectrometry. J Chromatogr B Analyt Technol Biomed Life Sci. 2017;1040:233–8. 10.1016/j.jchromb.2016.11.010. 27856194 10.1016/j.jchromb.2016.11.010 PMC5191936

[106] Kovarik M, Muthny T, Sispera L, Holecek M. Effects of β-hydroxy-β-methylbutyrate treatment in different types of skeletal muscle of intact and septic rats. J Physiol Biochem. 2010;66(4):311–9. 10.1007/s13105-010-0037-3. 20725872 10.1007/s13105-010-0037-3

[107] Holecek M, Muthny T, Kovarik M, Sispera L. Effect of beta-hydroxy-beta-methylbutyrate (HMB) on protein metabolism in whole body and in selected tissues. Food Chem Toxicol. 2009;47(1):255–9. 10.1016/j.fct.2008.11.021. 19056452 10.1016/j.fct.2008.11.021

[108] Suryawan A, Rudar M, Fiorotto ML, Davis TA. Differential regulation of mTORC1 activation by leucine and β-hydroxy-β-methylbutyrate in skeletal muscle of neonatal pigs. J Appl Physiol. 2020;128(2):286–95. 10.1152/japplphysiol.00332.2019. 31944890 10.1152/japplphysiol.00332.2019 PMC7052585

[109] Kimura K, Cheng XW, Inoue A, Hu L, Koike T, Kuzuya M. β-hydroxy-β-methylbutyrate facilitates PI3K/Akt-dependent mammalian target of rapamycin and FoxO1/3a phosphorylations and alleviates tumor necrosis factor α/interferon γ–induced MuRF-1 expression in C2C12 cells. Nutr Res. 2014;34(4):368–74. 10.1016/j.nutres.2014.02.003. 24774073 10.1016/j.nutres.2014.02.003

[110] Kornasio R, Riederer I, Butler-Browne G, Mouly V, Uni Z, Halevy O. Beta-hydroxy-beta-methylbutyrate (HMB) stimulates myogenic cell proliferation, differentiation and survival via the MAPK/ERK and PI3K/Akt pathways. Biochim Biophys Acta. 2009;1793(5):755–63. 10.1016/j.bbamcr.2008.12.017. 19211028 10.1016/j.bbamcr.2008.12.017

[111] Liu H, Liu R, Xiong Y, Li X, Wang X, Ma Y, et al. Leucine facilitates the insulin-stimulated glucose uptake and insulin signaling in skeletal muscle cells: involving mTORC1 and mTORC2. Amino Acids. 2014;46(8):1971–9. 10.1007/s00726-014-1752-9. 24806638 10.1007/s00726-014-1752-9

[112] Bear DE, Rooyackers O. HMB and leucine supplementation during critical illness and recovery. Curr Opin Clin Nutr Metab Care. 2022;25(2):88–92. 10.1097/mco.0000000000000809. 34937852 10.1097/MCO.0000000000000809

[113] Prado CM, Orsso CE, Pereira SL, Atherton PJ, Deutz NEP. Effects of β-hydroxy β-methylbutyrate (HMB) supplementation on muscle mass, function, and other outcomes in patients with cancer: a systematic review. J Cachexia Sarcopenia Muscle. 2022;13(3):1623–41. 10.1002/jcsm.12952. 35301826 10.1002/jcsm.12952 PMC9178154

[114] Cohen-Or M, Chapnik N, Froy O. B-hydroxy-β-methylbutyrate (HMB) leads to phospholipase D2 (PLD2) activation and alters circadian rhythms in myotubes. Food Funct. 2024;15(8):4389–98. 10.1039/d3fo04174c. 38563085 10.1039/d3fo04174c

[115] Kaczka P, Michalczyk MM, Jastrząb R, Gawelczyk M, Kubicka K. Mechanism of action and the effect of beta-hydroxy-beta-methylbutyrate (HMB) supplementation on different types of physical performance. J Hum Kinet. 2019;68:211–22. 10.2478/hukin-2019-0070. 31531146 10.2478/hukin-2019-0070 PMC6724588

[116] Zhang S, Tang Z, Zheng C, Zhong Y, Zheng J, Duan G, et al. Dietary beta-hydroxy-beta-methyl butyrate supplementation inhibits hepatic fat deposition via regulating gut microbiota in broiler chickens. Microorganisms. 2022;10(1):169. 10.3390/microorganisms10010169. 35056618 10.3390/microorganisms10010169 PMC8781658

[117] Wan M, Zheng C, Zheng J, Duan G, Yu J, Zhang P, et al. Different effects of dietary β-hydroxy-β-methylbutyrate on composition of fatty acid and free amino acid, and fatty metabolism in the different muscles of broilers. Poult Sci. 2023;102(10):103001. 10.1016/j.psj.2023.103001. 37604020 10.1016/j.psj.2023.103001 PMC10458338

[118] Zheng C, Song B, Duan Y, Zhong Y, Yan Z, Zhang S, et al. Dietary β-hydroxy-β-methylbutyrate improves intestinal function in weaned piglets after lipopolysaccharide challenge. Nutrition. 2020;78:110839. 10.1016/j.nut.2020.110839. 32540677 10.1016/j.nut.2020.110839

[119] Nilsen MS, Jersin R, Ulvik A, Madsen A, McCann A, Svensson PA, et al. 3-hydroxyisobutyrate, a strong marker of insulin resistance in type 2 diabetes and obesity that modulates white and brown adipocyte metabolism. Diabetes. 2020;69(9):1903–16. 10.2337/db19-1174. 32586980 10.2337/db19-1174 PMC7968520

[120] Jang C, Oh SF, Wada S, Rowe GC, Liu L, Chan MC, et al. A branched-chain amino acid metabolite drives vascular fatty acid transport and causes insulin resistance. Nat Med. 2016;22(4):421–6. 10.1038/nm.4057. 26950361 10.1038/nm.4057 PMC4949205

[121] Bjune MS, Lawrence-Archer L, Laupsa-Borge J, Sommersten CH, McCann A, Glastad RC, et al. Metabolic role of the hepatic valine/3-hydroxyisobutyrate (3-HIB) pathway in fatty liver disease. EBioMedicine. 2023;91:104569. 10.1016/j.ebiom.2023.104569. 37084480 10.1016/j.ebiom.2023.104569 PMC10148099

[122] Che L, Niu L, Liu L, Li M, Huo W, Deng H, et al. Reduction in within-litter variation of piglet birth weight through dietary supplementation of 3-hydroxyisobutyric acid in sows. Front Vet Sci. 2025;12:1646332. 10.3389/fvets.2025.1646332. 40895783 10.3389/fvets.2025.1646332 PMC12396199

[123] Prideaux M, Smargiassi A, Peng G, Brotto M, Robling AG, Bonewald LF. L-BAIBA synergizes with sub-optimal mechanical loading to promote new bone formation. JBMR Plus. 2023;7(6):e10746. 10.1002/jbm4.10746. 37283651 10.1002/jbm4.10746 PMC10241089

[124] Jung TW, Park HS, Choi GH, Kim D, Lee T. b-aminoisobutyric acid attenuates LPS-induced inflammation and insulin resistance in adipocytes through AMPK-mediated pathway. J Biomed Sci. 2018;25(1):27. 10.1186/s12929-018-0431-7. 29592806 10.1186/s12929-018-0431-7 PMC5875012

[125] Feng J, Wang X, Lu Y, Yu C, Wang X, Feng L. BAIBA involves in hypoxic training induced browning of white adipose tissue in obese rats. Front Physiol. 2022;13:882151. 10.3389/fphys.2022.882151. 35832480 10.3389/fphys.2022.882151 PMC9272788

[126] Minato T, Nakamura N, Saiki T, Miyabe M, Ito M, Matsubara T, et al. β-Aminoisobutyric acid, L-BAIBA, protects PC12 cells from hydrogen peroxide-induced oxidative stress and apoptosis via activation of the AMPK and PI3K/Akt pathway. IBRO Neurosci Rep. 2022;12:65–72. 10.1016/j.ibneur.2021.12.001. 35024688 10.1016/j.ibneur.2021.12.001 PMC8724974

[127] Crossland H, Smith K, Idris I, Phillips BE, Atherton PJ, Wilkinson DJ. Exploring mechanistic links between extracellular branched-chain amino acids and muscle insulin resistance: an in vitro approach. Am J Physiol Cell Physiol. 2020;319(6):C1151–7. 10.1152/ajpcell.00377.2020. 33026831 10.1152/ajpcell.00377.2020

[128] Leal Yepes FA, Mann S, Overton TR, Behling-Kelly E, Nydam DV, Wakshlag JJ. Hepatic effects of rumen-protected branched-chain amino acids with or without propylene glycol supplementation in dairy cows during early lactation. J Dairy Sci. 2021;104(9):10324–37. 10.3168/jds.2021-20265. 34176626 10.3168/jds.2021-20265

[129] Rivera CN, Kamer MM, Rivera ME, Watne RM, Macgowan TC, Wommack AJ, et al. Insulin resistance promotes extracellular BCAA accumulation without altering LAT1 content, independent of prior BCAA treatment in a myotube model of skeletal muscle. Mol Cell Endocrinol. 2023;559:111800. 10.1016/j.mce.2022.111800. 36270542 10.1016/j.mce.2022.111800

[130] Lynch CJ, Adams SH. Branched-chain amino acids in metabolic signalling and insulin resistance. Nat Rev Endocrinol. 2014;10(12):723–36. 10.1038/nrendo.2014.171. 25287287 10.1038/nrendo.2014.171 PMC4424797

[131] Abdualkader AM, Karwi QG, Lopaschuk GD, Al Batran R. The role of branched-chain amino acids and their downstream metabolites in mediating insulin resistance. J Pharm Pharm Sci. 2024;27:13040. 10.3389/jpps.2024.13040. 39007094 10.3389/jpps.2024.13040 PMC11239365

[132] Nishi K, Yoshii A, Abell L, Zhou B, Frausto R, Ritterhoff J, et al. Branched-chain keto acids inhibit mitochondrial pyruvate carrier and suppress gluconeogenesis in hepatocytes. Cell Rep. 2023;42(6):112641. 10.1016/j.celrep.2023.112641. 37310861 10.1016/j.celrep.2023.112641 PMC10592489

[133] Fahien LA, MacDonald MJ. The complex mechanism of glutamate dehydrogenase in insulin secretion. Diabetes. 2011;60(10):2450–4. 10.2337/db10-1150. 21948999 10.2337/db10-1150 PMC3178282

[134] Arrieta-Cruz I, Su Y, Gutiérrez-Juárez R. Suppression of endogenous glucose production by isoleucine and valine and impact of diet composition. Nutrients. 2016;8(2):79. 10.3390/nu8020079. 26891318 10.3390/nu8020079 PMC4772043

[135] Duan Y, Li F, Wang W, Guo Q, Wen C, Yin Y. Alteration of muscle fiber characteristics and the AMPK-SIRT1-PGC-1α axis in skeletal muscle of growing pigs fed low-protein diets with varying branched-chain amino acid ratios. Oncotarget. 2017;8(63):107011–21. 10.18632/oncotarget.22205. 29291007 10.18632/oncotarget.22205 PMC5739792

[136] Zhang S, Yang Q, Ren M, Qiao S, He P, Li D, et al. Effects of isoleucine on glucose uptake through the enhancement of muscular membrane concentrations of GLUT1 and GLUT4 and intestinal membrane concentrations of Na^+^/glucose co-transporter 1 (SGLT-1) and GLUT2. Br J Nutr. 2016;116(4):593–602. 10.1017/s0007114516002439. 27464458 10.1017/S0007114516002439

[137] Li T, Zhang Z, Kolwicz SC Jr, Abell L, Roe ND, Kim M, et al. Defective branched-chain amino acid catabolism disrupts glucose metabolism and sensitizes the heart to ischemia-reperfusion injury. Cell Metab. 2017;25(2):374–85. 10.1016/j.cmet.2016.11.005. 28178567 10.1016/j.cmet.2016.11.005 PMC5301464

[138] He L, Gomes AP, Wang X, Yoon SO, Lee G, Nagiec MJ, et al. mTORC1 promotes metabolic reprogramming by the suppression of GSK3-dependent Foxk1 phosphorylation. Mol Cell. 2018;70(5):949-60.e4. 10.1016/j.molcel.2018.04.024. 29861159 10.1016/j.molcel.2018.04.024 PMC6591025

[139] Geng L, Liao B, Jin L, Yu J, Zhao X, Zhao Y, et al. B-klotho promotes glycolysis and glucose-stimulated insulin secretion via GP130. Nat Metab. 2022;4(5):608–26. 10.1038/s42255-022-00572-2. 35551509 10.1038/s42255-022-00572-2

[140] Trefts E, Shaw RJ. AMPK: restoring metabolic homeostasis over space and time. Mol Cell. 2021;81(18):3677–90. 10.1016/j.molcel.2021.08.015. 34547233 10.1016/j.molcel.2021.08.015 PMC8549486

[141] Dalle Pezze P, Ruf S, Sonntag AG, Langelaar-Makkinje M, Hall P, Heberle AM, et al. A systems study reveals concurrent activation of AMPK and mTOR by amino acids. Nat Commun. 2016;7:13254. 10.1038/ncomms13254. 10.1038/ncomms13254 PMC5121333 27869123

[142] Melick CH, Jewell JL. Regulation of mTORC1 by upstream stimuli. Genes. 2020;11(9):989. 10.3390/genes11090989. 32854217 10.3390/genes11090989 PMC7565831

[143] Hinkle JS, Rivera CN, Vaughan RA. AICAR stimulates mitochondrial biogenesis and BCAA catabolic enzyme expression in C2C12 myotubes. Biochimie. 2022;195:77–85. 10.1016/j.biochi.2021.11.004. 34798200 10.1016/j.biochi.2021.11.004

[144] Herzig S, Shaw RJ. AMPK: guardian of metabolism and mitochondrial homeostasis. Nat Rev Mol Cell Biol. 2018;19(2):121–35. 10.1038/nrm.2017.95. 28974774 10.1038/nrm.2017.95 PMC5780224

[145] Salinas-Rubio D, Tovar AR, Noriega LG. Emerging perspectives on branched-chain amino acid metabolism during adipocyte differentiation. Curr Opin Clin Nutr Metab Care. 2018;21(1):49–57. 10.1097/mco.0000000000000429. 29035970 10.1097/MCO.0000000000000429

[146] Rong S, Xia M, Vale G, Wang S, Kim CW, Li S, et al. DGAT2 inhibition blocks SREBP-1 cleavage and improves hepatic steatosis by increasing phosphatidylethanolamine in the ER. Cell Metab. 2024;36(3):617-29.e7. 10.1016/j.cmet.2024.01.011. 38340721 10.1016/j.cmet.2024.01.011 PMC10939742

[147] Düvel K, Yecies JL, Menon S, Raman P, Lipovsky AI, Souza AL, et al. Activation of a metabolic gene regulatory network downstream of mTOR complex 1. Mol Cell. 2010;39(2):171–83. 10.1016/j.molcel.2010.06.022. 20670887 10.1016/j.molcel.2010.06.022 PMC2946786

[148] Chen Q, Wang C, Sun Y, Chen S, Zhou J, Han T, et al. An integrated analysis of transcriptome and metabolome reveals three BCAAs relieve lipid accumulation by inhibiting lipid synthesis and promoting lipid oxidation in the liver of largemouth bass (*Micropterus salmoides*). Aquaculture. 2024;581:740384. 10.1016/j.aquaculture.2023.740384.

[149] Liang C, Curry BJ, Brown PL, Zemel MB. Leucine modulates mitochondrial biogenesis and SIRT1-AMPK signaling in C2C12 myotubes. J Nutr Metab. 2014;2014:239750. 10.1155/2014/239750. 25400942 10.1155/2014/239750 PMC4220583

[150] Jian H, Li R, Huang X, Li J, Li Y, Ma J, et al. Branched-chain amino acids alleviate NAFLD via inhibiting de novo lipogenesis and activating fatty acid β-oxidation in laying hens. Redox Biol. 2024;77:103385. 10.1016/j.redox.2024.103385. 39426289 10.1016/j.redox.2024.103385 PMC11536022

[151] Zhang F, Zhao S, Yan W, Xia Y, Chen X, Wang W, et al. Branched chain amino acids cause liver injury in obese/diabetic mice by promoting adipocyte lipolysis and inhibiting hepatic autophagy. EBioMedicine. 2016;13:157–67. 10.1016/j.ebiom.2016.10.013. 27843095 10.1016/j.ebiom.2016.10.013 PMC5264279

[152] Guo F, Cavener DR. The GCN2 eIF2α kinase regulates fatty-acid homeostasis in the liver during deprivation of an essential amino acid. Cell Metab. 2007;5(2):103–14. 10.1016/j.cmet.2007.01.001. 10.1016/j.cmet.2007.01.001 17276353

[153] Du Y, Meng Q, Zhang Q, Guo F. Isoleucine or valine deprivation stimulates fat loss via increasing energy expenditure and regulating lipid metabolism in WAT. Amino Acids. 2012;43(2):725–34. 10.1007/s00726-011-1123-8. 22016194 10.1007/s00726-011-1123-8

[154] Habibi M, Shili C, Sutton J, Goodarzi P, Maylem ER, Spicer L, et al. Branched-chain amino acids partially recover the reduced growth of pigs fed with protein-restricted diets through both central and peripheral factors. Anim Nutr. 2021;7(3):868–82. 10.1016/j.aninu.2021.02.002. 34632118 10.1016/j.aninu.2021.02.002 PMC8484988

[155] Zhang S, Qiao S, Ren M, Zeng X, Ma X, Wu Z, et al. Supplementation with branched-chain amino acids to a low-protein diet regulates intestinal expression of amino acid and peptide transporters in weanling pigs. Amino Acids. 2013;45(5):1191–205. 10.1007/s00726-013-1577-y. 23990159 10.1007/s00726-013-1577-y

[156] Zheng L, Wei H, Cheng C, Xiang Q, Pang J, Peng J. Supplementation of branched-chain amino acids to a reduced-protein diet improves growth performance in piglets: involvement of increased feed intake and direct muscle growth-promoting effect. Br J Nutr. 2016;115(12):2236–45. 10.1017/s0007114516000842. 27079773 10.1017/S0007114516000842

[157] Tian M, Heng J, Song H, Shi K, Lin X, Chen F, et al. Dietary branched-chain amino acids regulate food intake partly through intestinal and hypothalamic amino acid receptors in piglets. J Agric Food Chem. 2019;67(24):6809–18. 10.1021/acs.jafc.9b02381. 31134808 10.1021/acs.jafc.9b02381

[158] Gerspach AC, Steinert RE, Schönenberger L, Graber-Maier A, Beglinger C. The role of the gut sweet taste receptor in regulating GLP-1, PYY, and CCK release in humans. Am J Physiol Endocrinol Metab. 2011;301(2):E317–25. 10.1152/ajpendo.00077.2011. 21540445 10.1152/ajpendo.00077.2011

[159] Chotechuang N, Azzout-Marniche D, Bos C, Chaumontet C, Gausserès N, Steiler T, et al. mTOR, AMPK, and GCN2 coordinate the adaptation of hepatic energy metabolic pathways in response to protein intake in the rat. Am J Physiol Endocrinol Metab. 2009;297(6):E1313–23. 10.1152/ajpendo.91000.2008. 19738034 10.1152/ajpendo.91000.2008

[160] Maurin A-C, Benani A, Lorsignol A, Brenachot X, Parry L, Carraro V, et al. Hypothalamic eIF2α signaling regulates food intake. Cell Rep. 2014;6(3):438–44. 10.1016/j.celrep.2014.01.006. 24485657 10.1016/j.celrep.2014.01.006 PMC4876923

[161] Zhang S, Chu L, Qiao S, Mao X, Zeng X. Effects of dietary leucine supplementation in low crude protein diets on performance, nitrogen balance, whole-body protein turnover, carcass characteristics and meat quality of finishing pigs. Anim Sci J. 2016;87(7):911–20. 10.1111/asj.12520. 26597995 10.1111/asj.12520

[162] Luo Y, Zhang X, Zhu Z, Jiao N, Qiu K, Yin J. Surplus dietary isoleucine intake enhanced monounsaturated fatty acid synthesis and fat accumulation in skeletal muscle of finishing pigs. J Anim Sci Biotechnol. 2018;9:88. 10.1186/s40104-018-0306-5. 10.1186/s40104-018-0306-5 PMC6302484 30598820

[163] Xu D, Wang Y, Jiao N, Qiu K, Zhang X, Wang L, et al. The coordination of dietary valine and isoleucine on water holding capacity, pH value and protein solubility of fresh meat in finishing pigs. Meat Sci. 2020;163:108074. 10.1016/j.meatsci.2020.108074. 32036285 10.1016/j.meatsci.2020.108074

[164] Wu G. Dietary requirements of synthesizable amino acids by animals: a paradigm shift in protein nutrition. J Anim Sci Biotechnol. 2014;5:34. 10.1186/2049-1891-5-34. 10.1186/2049-1891-5-34 PMC4082180 24999386

[165] Oluwabiyi CT, Song Z. Branched-chain amino acids supplementation in low-protein broiler diets: a review. Anim Feed Sci Tech. 2024;318:116114. 10.1016/j.anifeedsci.2024.116114.

[166] Greenhalgh S, Macelline SP, Chrystal PV, Liu SY, Selle PH. Elevated branched-chain amino acid inclusions generate distinctly divergent growth performance responses in broiler chickens offered wheat- and/or sorghum-based, reduced-crude protein diets. Anim Feed Sci Tech. 2022;292:115446. 10.1016/j.anifeedsci.2022.115446.

[167] Goo D, Singh AK, Choi J, Sharma MK, Paneru D, Lee J, et al. Different dietary branched-chain amino acid ratios, crude protein levels, and protein sources can affect the growth performance and meat yield in broilers. Poult Sci. 2024;103(12):104313. 10.1016/j.psj.2024.104313. 39357235 10.1016/j.psj.2024.104313 PMC11474198

[168] Ospina-Rojas IC, Pozza PC, Rodrigueiro RJB, Gasparino E, Khatlab AS, Murakami AE. High leucine levels affecting valine and isoleucine recommendations in low-protein diets for broiler chickens. Poult Sci. 2020;99(11):5946–59. 10.1016/j.psj.2020.08.053. 33142512 10.1016/j.psj.2020.08.053 PMC7647919

[169] Ma S, Zhang K, Shi S, Li X, Che C, Chen P, et al. Low-protein diets supplemented with isoleucine alleviate lipid deposition in broilers through activating 5′ adenosine monophosphate-activated protein kinase and janus kinase 2/signal transducer and activator of transcription 3 signaling pathways. Poult Sci. 2023;102(3):102441. 10.1016/j.psj.2022.102441. 36599221 10.1016/j.psj.2022.102441 PMC9823210

[170] Lv X, Jiang A, Hua J, Liu Z, Yan Q, Tang S, et al. Long-term leucine supplementation increases body weight in goats by controlling appetite and muscle protein synthesis under protein-restricted conditions. Anim Nutr. 2024;20:404–18. 10.1016/j.aninu.2024.09.005. 10.1016/j.aninu.2024.09.005 PMC11872668 40034461

[171] Xu W, Kenéz Á, Mann S, Overton TR, Wakshlag JJ, Nydam DV, et al. Effects of dietary branched-chain amino acid supplementation on serum and milk metabolome profiles in dairy cows during early lactation. J Dairy Sci. 2022;105(10):8497–508. 10.3168/jds.2022-21892. 35965128 10.3168/jds.2022-21892

[172] Mao H, Wang C, Yu Z. Dietary leucine supplementation enhances the health of early weaned Hu lambs. Anim Feed Sci Tech. 2019;247:248–54. 10.1016/j.anifeedsci.2018.11.020.

[173] Curtis RV, Kim JJM, Doelman J, Cant JP. Maintenance of plasma branched-chain amino acid concentrations during glucose infusion directs essential amino acids to extra-mammary tissues in lactating dairy cows. J Dairy Sci. 2018;101(5):4542–53. 10.3168/jds.2017-13236. 29477518 10.3168/jds.2017-13236

[174] Doelman J, Kim JJ, Carson M, Metcalf JA, Cant JP. Branched-chain amino acid and lysine deficiencies exert different effects on mammary translational regulation. J Dairy Sci. 2015;98(11):7846–55. 10.3168/jds.2015-9819. 26342977 10.3168/jds.2015-9819

[175] Appuhamy JA, Knapp JR, Becvar O, Escobar J, Hanigan MD. Effects of jugular-infused lysine, methionine, and branched-chain amino acids on milk protein synthesis in high-producing dairy cows. J Dairy Sci. 2011;94(4):1952–60. 10.3168/jds.2010-3442. 21426986 10.3168/jds.2010-3442

[176] Chen J, Lei XJ, Wang L, Zhang YL, Wang DD, Zhao LC, et al. Effects of rumen-protected leucine on production performance and starch digestion in the small intestine of lactating goats. Anim Feed Sci Technol. 2022;287:115270. 10.1016/j.anifeedsci.2022.115270.

[177] Leal Yepes FA, Mann S, Overton TR, Ryan CM, Bristol LS, Granados GE, et al. Effect of rumen-protected branched-chain amino acid supplementation on production- and energy-related metabolites during the first 35 days in milk in Holstein dairy cows. J Dairy Sci. 2019;102(6):5657–72. 10.3168/jds.2018-15508. 30928273 10.3168/jds.2018-15508

[178] Jorge-Smeding E, Bonnet M, Renand G, Taussat S, Graulet B, Ortigues-Marty I, et al. Common and diet-specific metabolic pathways underlying residual feed intake in fattening Charolais yearling bulls. Sci Rep. 2021;11:24346. 10.1038/s41598-021-03678-x. 10.1038/s41598-021-03678-x PMC8692463 34934071

[179] Jorge-Smeding E, Polakof S, Bonnet M, Durand S, Centeno D, Pétéra M, et al. Untargeted metabolomics confirms the association between plasma branched chain amino acids and residual feed intake in beef heifers. PLoS ONE. 2022;17(11):e0277458. 10.1371/journal.pone.0277458. 36445891 10.1371/journal.pone.0277458 PMC9707789

[180] Duarte DAS, Newbold CJ, Detmann E, Silva FF, Freitas PHF, Veroneze R, et al. Genome-wide association studies pathway-based meta-analysis for residual feed intake in beef cattle. Anim Genet. 2019;50(2):150–3. 10.1111/age.12761. 30644110 10.1111/age.12761

